# Investigating seasonal patterns in enteric infections: a systematic review of time series methods

**DOI:** 10.1017/S0950268822000243

**Published:** 2022-02-14

**Authors:** Ryan B. Simpson, Alexandra V. Kulinkina, Elena N. Naumova

**Affiliations:** 1 Tufts University Friedman School of Nutrition Science and Policy, Boston, MA 02111, USA; 2 Swiss Tropical and Public Health Institute, Basel, Switzerland; 3 University of Basel, Basel, Switzerland

**Keywords:** Foodborne infection, gastrointestinal infection, reproducibility, season, seasonality, time series analysis, waterborne infection

## Abstract

Foodborne and waterborne gastrointestinal infections and their associated outbreaks are preventable, yet still result in significant morbidity, mortality and revenue loss. Many enteric infections demonstrate seasonality, or annual systematic periodic fluctuations in incidence, associated with climatic and environmental factors. Public health professionals use statistical methods and time series models to describe, compare, explain and predict seasonal patterns. However, descriptions and estimates of seasonal features, such as peak timing, depend on how researchers define seasonality for research purposes and how they apply time series methods. In this review, we outline the advantages and limitations of common methods for estimating seasonal peak timing. We provide recommendations improving reporting requirements for disease surveillance systems. Greater attention to how seasonality is defined, modelled, interpreted and reported is necessary to promote reproducible research and strengthen proactive and targeted public health policies, intervention strategies and preparedness plans to dampen the intensity and impacts of seasonal illnesses.

## Introduction

Foodborne and waterborne gastrointestinal infections are caused by the consumption of contaminated food and water, respectively [1, 2]. Food contamination often occurs due to poor health or hygiene practices during food production, processing, distribution or consumption, or cross-contamination between food products [1–3]. Waterborne illnesses and outbreaks are often attributed to ageing infrastructure and severe weather events [4–6]. Outbreaks are commonly defined as the occurrences of two or more infections caused by the same source [1–3]. Infection incubation periods range from a few hours to many months, causing high volumes and broad geographic extent of infected persons per outbreak. This, combined with the habitude of human food and water consumption, the globalisation of food supply chains, and the frequent handling or storage of food and water before consumption, makes sources of gastrointestinal infections difficult to identify and control [3, 7–9].

Public health professionals often use time series analyses, or a collection of methods to describe, explain and predict temporal processes, to assess the patterns of infections [10–12]. Time series analyses examine population-based likelihoods of infections as a function of time and time-varying factors [13]. These methods decompose the temporal distribution of infections into three components: (i) trend or general incidence fluctuation over time; (ii) seasonality or systematic periodic fluctuation in incidence generally observed over 1 year; and (iii) change in incidence driven by other periodic, sporadic or random events [14]. Seasonality can be further characterised by three main features: (i) peak (and nadir) timing or when incidence reaches its maximum (and minimum); (ii) amplitude or the difference in incidence between seasonal peaks and nadirs; and (iii) duration or the time interval when incidence rises above a specified threshold [12, 15]. Most modelling studies detect the presence of seasonality by characterizing peak timing.

Precise estimation of peak timing helps identify differences in seasonal patterns by pathogen, subpopulation and geographic location [12]. Synchronisation of pathogens' peaks suggests possible co-infections and shared food/water- or environmental-drivers of infection [12, 16, 17]. Lags between peaks of infections and their drivers, best assessed with more granular temporal data, inform forecasts of peak incidence for early outbreak warnings [12, 18, 19]. Better characterisation of seasonal patterns in diseases and exposures contributes to refinements of research hypotheses by identifying non-seasonal drivers such as stagnant water, agricultural runoff, livestock migration or sanitation practices [20–22]. Accurate peak timing estimates by subpopulation and geographic location identify when and where vulnerable populations are at the highest risk of infection [12].

While the concept of seasonality may seem straightforward, its characterisation and quantification are complex. Researchers often detect seasonality and estimate seasonal peak timing by comparing average incidence or cumulative infections by season [23]. Alternatively, researchers can describe seasonality as a continuous temporal process. Researchers model temporal patterns using data-driven methods based on smoothing or polynomial, periodic and other non-linear functions [14]. Researchers detect seasonality with a formal statistical test of periodicity or by assessing model fit [23, 24]. These approaches and their methods have advantages and limitations that have been thoroughly explored for modelling seasonal respiratory infections like influenza and pneumonia [25–28]. However, differences in terminology for defining seasonality and limitations of methods for estimating seasonal peak timing remain largely underexplored within literature related to gastrointestinal infections.

In this systematic review, we demonstrate differences in applied definitions of seasonality when modelling, interpreting and reporting gastrointestinal infections. We extracted and reviewed original research articles that detected and estimated the seasonality features of gastrointestinal infections in humans using disease surveillance systems or hospital health records. We describe advantages and limitations of statistical methods for detecting, estimating and comparing seasonal peak timing. Standardised terminology and reporting requirements can be extended to the field of infectious disease epidemiology.

## Methods

### Literature search

We conducted a search of published literature in peer-reviewed journals using the National Library of Medicine's National Center for Biotechnology Information bibliographic database *PubMed*. This database identified publications spanning many disciplines related to enteric infections including nutritional, agricultural, environmental, public health, zoonotic and microbiological sciences. *PubMed*'s emphasis on circulating research with innovative analytical approaches prioritised publications that thoroughly reported on statistical methodologies [29].

We used no publishing date restrictions. We conducted our first search on 02 March 2019 with follow-up searches on 18 July 2019, 20 December 2020 and 22 July 2021. For each search, we included the following search terms forming four categories:

- *Disease aetiology*: ‘foodborne’, ‘food borne’, ‘food-borne’, ‘waterborne’, ‘water borne’, ‘water-borne’ and ‘gastroenteritis’;
- *Notifiable gastrointestinal infections*: ‘*Campylobacter*’, ‘campylobacteriosis’, ‘*Salmonella*’, ‘salmonellosis’, ‘*Vibrio*’, ‘vibriosis’, ‘cholera’, ‘*Listeria*’, ‘listeriosis’, ‘*Cryptosporidium*’, ‘cryptosporidiosis’, ‘*Shigella*’, ‘shigellosis’, ‘*Cyclospora*’, ‘cyclosporiasis’, ‘*Escherichia*’, ‘e. coli’, ‘*Yersinia*’, ‘yersiniosis’, ‘norovirus’, ‘*Giardia*’, ‘giardiasis’, ‘rotavirus’ and ‘rotaviral’;
- *Case definitions*: ‘illness’, ‘infection’, ‘incidence’, ‘rate’ and ‘outbreak’; and
- *Key terms*: ‘season’, ‘seasonal’, ‘seasonality’ and ‘peak’.

We selected pathogens using nationally notifiable enteric infections reported by the US Centers for Disease Control and Prevention's (CDC's) Foodborne Diseases Active Surveillance Network (FoodNet) and National Outbreak Reporting System (NORS) [9, 30–33]. These notifiable infections represented internationally monitored enteric infections [34, 35].

### Exclusion criteria and study abstraction

**Fig. 1.**
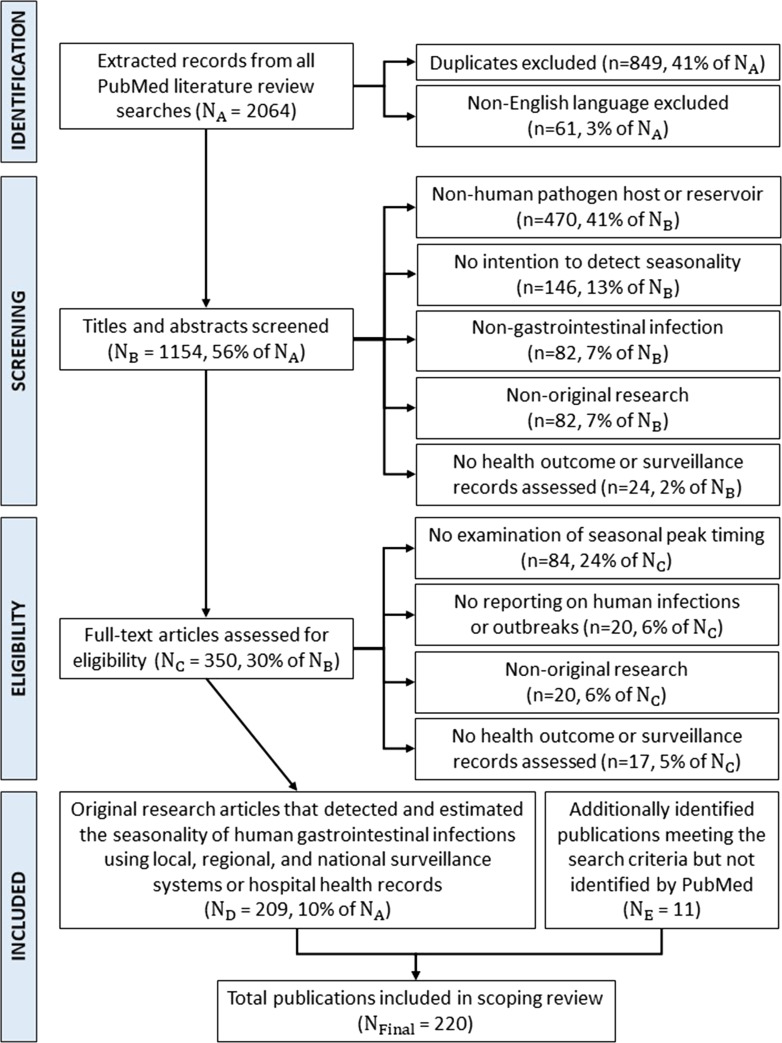
PRISMA flow diagram detailing the identification, screening, eligibility and inclusion of articles for our systematic review. Included studies (*n* = 220) were original research articles that detected and estimated the seasonality of human gastrointestinal infections using local, regional and national surveillance systems or hospital health records.

Our search yielded 2064 publications (Fig. 1). First, we excluded non-English publications (*n* = 61) and duplicates (*n* = 849). Next, we reviewed the abstracts of the remaining 1154 publications and excluded those with: (i) animal, plant or water hosts/reservoirs (*n* = 470); (ii) <1 year of data, case control studies or ecologic studies (*n* = 146); (iii) non-gastrointestinal infections (*n* = 82); (iv) literature reviews, editorials or viewpoints (*n* = 82); and (v) studies examining antimicrobial resistance, genetic diversity, and economic burdens of illness or using simulation-generated data (*n* = 24). We conducted a full-text review on the remaining 350 publications and excluded publications with no estimation of seasonal peak timing (*n* = 84), no reporting on human illnesses or outbreaks (*n* = 20), non-original research (*n* = 20), and no health outcome or surveillance records assessed (*n* = 17).

We reviewed references of the 209 extracted publications for additional manuscripts meeting the above inclusion criteria but not identified in our initial search (Fig. 1). We also reviewed featured publications on FoodNet and NORS websites [36, 37]. We identified 11 additional publications meeting our search criteria that were not identified in our *PubMed* search. We exported and reviewed all 220 citations using EndNote version X7.7.1 software. We report all citations in Supplementary Table S1, which are referenced below using a citation identifier (CID). We designed and created all visualisations using PowerPoint version 14.3.6 and R version 3.6.2 software.

### Structure of findings

First, we describe study objectives and rationale for investigating seasonality. Next, we discuss two approaches for detecting and estimating seasonality features: (i) comparisons of discrete time intervals; and (ii) construction of seasonal curves. For comparisons by discrete time intervals, we compare methods used when defining two and four seasons or the use of discrete calendar months. For constructed seasonal curves, we compare common modelling techniques including average smoothers, cubic splines, seasonal trend decomposition (STL), seasonal autoregressive integrated moving averages (SARIMA), harmonic regression models and spectral analyses. For each method, we outline advantages and limitations for detecting seasonality and estimating seasonal peak timing.

## Results

### Motivation and rationale for investigating seasonality

Studies investigating seasonality aimed to: (i) describe trends in infections over time; (ii) compare trends and seasonality features by pathogen, subpopulation, geographic location or other risk factors; and/or (iii) explain associations between seasonal infections and their environmental drivers. Descriptive studies often defined seasonality using discrete seasons or calendar months (Table 1). Comparative and explanatory studies examined the seasonality of common pathogens like *Salmonella* and *Campylobacter* by constructing seasonal curves. For example, *Norovirus* studies compared seasonal peak timing by strain/subtype while *Escherichia coli*, *Cryptosporidium* and *Vibrio* studies compared peak timing across geographic locations or subpopulations using both discrete seasons and seasonal curves (Table 1).

**Table 1.** A summary of time series methods for describing, comparing and explaining the seasonality of the 14 most cited gastrointestinal infections from our review

Pathogen	Total citations	Discrete seasons			Seasonal curves as continuous processes					
		Two seasons	Four seasons	Monthly records	Average smoothers	Cubic splines	STL models	SARIMA models	Harmonic regressions	Spectral analyses
*Salmonella*	66	5	25	22	1			2	12	
*Campylobacter*	45	2	20	9	1	1	1		9	2
Gastroenteritis	38	1	15	15		1		2	4	
*Vibrio*	20	2	7	4		1		2	4	
*Norovirus*	19	3	9	6						
*E. coli*	18		14	1			1		2	
*Cryptosporidium*	17	2	1	10		1			3	
*Yersinia*	11		7	3					1	
*Shigella*	11		7	2					2	
*Giardia*	10	1	3	3				1	2	
*Listeria*	9		6	2					1	
*Cyclospora*	6	1	1	4					1	
Rotavirus	6	2	1						3	1
*Clostridium*	2			2						
Total	215	17	76	74	2	4	2	7	31	2

We ranked pathogens in descending order by total citations. We divided methods by comparisons of discrete seasons and the construction of seasonal curves. Discrete seasons methods included comparisons by two seasons, four seasons or calendar months. Seasonal curve methods included average smoothers, cubic splines, seasonal trend decomposition (STL), seasonal autoregressive integrated moving average (SARIMA) models, harmonic regression models and spectral analyses. Column and row totals are less than the sum of all rows and columns, respectively, as many publications investigated multiple pathogens and used multiple methodologies to describe, compare and explain seasonality features.

#### Describing trends in infections over time

Descriptive studies often reported linear fluctuations in mean and median incidence over time or the sum and percentage of infections by consecutive seasons or months (CID 1–11). Studies frequently estimated seasonal peak timing by identifying the calendar month or season with the highest sum, percentage or average incidence of infections (CID 12–18). Study rationales aimed to understand trends in infection severity for creating, modifying or evaluating public health interventions. Researchers stated that linear trends and seasonal peak timing estimates informed when, where and for which pathogen health safety policies were most needed (CID 13, 19–21). Researchers equated reductions in infections or dampening of seasonal peak intensities to positive benefits of enacted health policies or programmes (CID 12, 22).

Additional study rationales included reviewing surveillance system capacity and preparing public health institutions for seasonal hospitalisation peaks (CID 23–30). Many early studies described trends and seasonality features to assist public health laboratories in detecting, monitoring and tracking infections (CID 23–28). Peak timing estimates aided public health institutions in: (i) managing personnel and supplies (CID 30); and (ii) communicating risks of gastrointestinal infections to travellers or residents in high-risk areas during outbreak seasons (CID 29). Studies often emphasised the importance of improving the spatial and temporal resolution of surveillance records to permit more precise, accurate and reliable estimation of seasonality features.

#### Comparing seasonal peak timing by subgroups

Comparative studies estimated seasonality features by pathogen strain, subpopulation, geographic location or other risk factors (CID 31–38). Surveillance systems collected and reported cases by pathogen subtype as serotyping technologies became available. Many studies compared *Norovirus* and *Salmonella* strains to demonstrate the utility of advanced laboratory testing capabilities (CID 1, 4, 39–43). Studies identified which strains were most severe, required greatest allocation of laboratory resources and needed continued surveillance during outbreak seasons (CID 1, 42, 43). Descriptive assessments of seasonal peak timing by enteric species' subtypes informed the circulation patterns of common strains and their epidemiological or clinical characteristics, which can improve the specificity of lab methods used for pathogen detection (CID 39). Researchers also compared peak timing by strain to forecast disease burden in upcoming outbreak seasons (CID 4, 40, 41).

Comparisons by subpopulation and geographic location identified who and where was at the highest risk of infection (CID 44–50). Study rationales aimed to provide information for developing infection prevention and management guidelines in future outbreaks (CID 46–48, 51). Studies investigating subpopulations compared trends and seasonality by sex, age group or place where infection was acquired (domestic- *vs.* travel-related) (CID 46, 52). Geographical comparisons attempted to identify the hotspots of infection vulnerable to multi-county or multi-state outbreaks (CID 47, 53). Many comparison studies lacked formal statistical tests for detecting significant peak timing differences.

#### Explaining associations between infections and risk factors

Few studies investigated associations between seasonal infections and environmental factors such as ambient temperature, precipitation and relative humidity (CID 54–60). These studies often extracted daily or weekly time series data and explored seasonal patterns using harmonic regression models (CID 18, 37, 61–65). Studies quantified associations between health and environmental variables using lags, which varied in length according to data's temporal resolution (CID 66–76). Study findings informed early warning forecasts or evaluated the effect of extreme weather events on the amplification or dampening of incidence in human infections (CID 55, 58, 64, 66, 67, 69). Researchers noted that their understanding of associations between environmental drivers and human infections improved the reliability of peak timing forecasts (CID 55, 66, 68). Studies encouraged continued examination of associations between environmental drivers and gastrointestinal infections considering ongoing climate changes globally (CID 58, 59, 77).

### Describing seasonality with discrete seasons

Most studies detected seasonality and examined seasonality features using discrete seasons (Table 1). Researchers defined seasons either analytically using surveillance data (e.g. high/low incidence) (CID 78–82) or using external biological, environmental, physical, physiological or other assumptions (e.g. wet/dry, warm/cool, summer/fall/winter/spring) (CID 3, 18, 33, 37, 80, 81, 83–86). This approach closely reflected layman definitions of season: ‘a period normally characterised by a particular kind of weather’ or ‘a period marked by special activity especially in some field’ [38]. Seasons had equal or unequal lengths and were defined by pre-determined dates or known patterns of disease incidence. To detect seasonal peak timing, researchers identified one season with significantly higher incidence than all others. While we only describe applications of two and four seasons below, studies might also define three, five or more seasons [39–42].

#### Two seasons

Studies often defined two seasons with equal 6-month lengths that spanned either a single calendar year such as two semesters (e.g. January–June *vs.* July–December) or two adjacent calendar years (e.g. October–March *vs.* April–September) (Supplementary Table S2; CID 48, 78, 79). Studies defined unequal season lengths when analytically deriving seasons based on incidence, which often consisted of a ~3-month high-incidence season compared to a ~9-month low-incidence season (CID 58, 80, 81). Unequal season lengths varied by genus, strain or serotype of the pathogen(s) assessed (CID 81).

Studies defining seasons with environmental factors used meteorological patterns like coolness and warmness, wetness and dryness, or their combination. Cool and warm seasons varied by climate region and hemisphere (CID 37, 87). Some studies defined seasons by extreme temperature events, such as heatwaves, whose dates varied by annual cycle (CID 84). Other studies defined cool and warm seasons by aggregating times-of-the-year with similar temperatures such as spring/summer and fall/winter (CID 79). Researchers defined wet and dry seasons using precipitation or relative humidity and often examined associations between flooding events and incidence (CID 58, 60, 88, 89). These studies aimed to investigate the effects of seasonal surface water flooding dynamics on human health (CID 88, 89).

Statistical analyses for detecting seasonality with two seasons are computationally straightforward. However, in the absence of formal comparison tests, differences in incidence by season could be spurious. While studies used terminology like ‘seasonal peaks’ or ‘greater incidence’ to imply formal inferences, seasonality was undetermined without test results. Studies applied pairwise Student's *t*-tests, Mann–Whitney rank-sum tests or *χ*^2^-tests to compare average incidence, median incidence or cumulative infections, respectively (CID 37, 79, 89). Statistical power depended on the number of study years assessed as seasons occurred only once per year.

Recent studies conducted formal comparisons using binary variables within logistic regression models (CID 37, 48, 58, 60, 80, 82–84). Models compared seasons using odds ratio (OR) and incidence rate ratio (IRR) measures of association. Studies applying these models had the advantage of adjusting for additional confounding factors expected to influence seasonality (CID 80, 82–84).

**Fig. 2.**
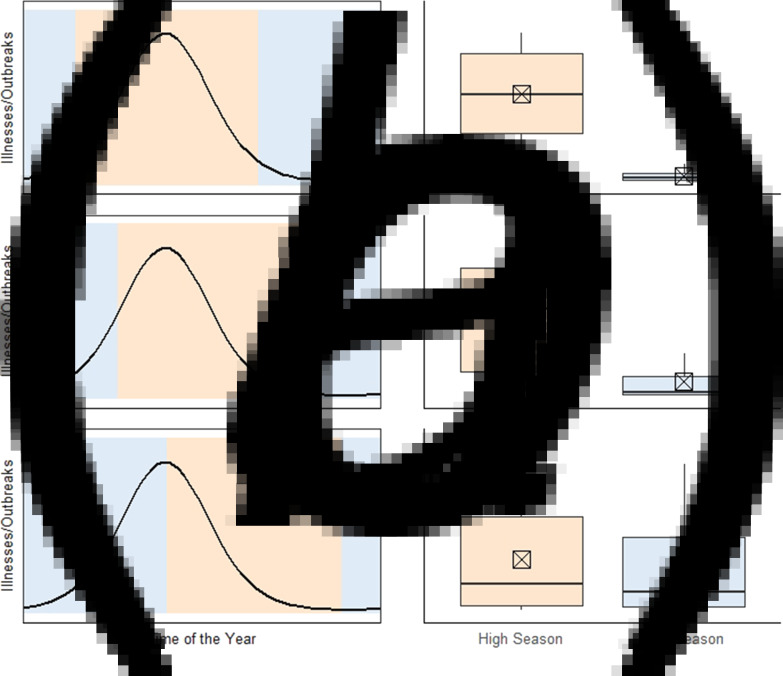
An illustration of detecting seasonality and estimating seasonal peak timing using two discrete seasons. Scenarios include (a) when peak and nadir timing align with the centre of each season, as expected for incidence-based definitions of seasons; (b) when peak and nadir timing is shifted from the centre of each season; and (c) when peak timing aligns with the boundary between seasons and results in substantial misclassification bias.

When comparing two seasons, peaks and nadirs ideally fall at the centre of each season in each year (Fig. 2a). Equal season lengths neatly divide time points of the highest and lowest incidence into each season. However, many enteric infections exhibit irregularities when long periods of low incidence alternate with short bursts of infections, or when extended periods of high incidence are replaced with intermittent declines. This complicates analytically deriving season intervals and can lead to misclassification when *a priori* assigned seasons align poorly (Fig. 2b) or not at all (Fig. 2c) with actual data. Poor alignment will result in more similar average or median incidence between seasons, decreasing a researcher's ability to detect seasonality. If peak timing falls near or at the boundary between seasons, differences will be indistinguishable, and researchers will not detect seasonality, resulting in misclassification bias (Fig. 2c).

Seasons defined using exogenous environmental or biological assumptions could align well with seasonal peaks or nadirs and demonstrate strong correlations as in Figure 2a. However, environmental seasons and infection seasonality could be misaligned resulting in low (Fig. 2b) or no correlation (Fig. 2c). Low or no correlation does not indicate the absence of disease seasonality but rather a potential lag in peak timing from the centre of the season compared to an environmental factor's seasonal peak.

In general, the precision for estimating seasonal peak timing is low using this method. Coarse aggregation of seasons suggests that peak timing could occur anytime within a ~6-month interval. This inhibits informative public health policies and prevents investigating possible trends, drifts or instantaneous shifts of seasonal curves over time. Data compression also underutilises daily, weekly and monthly surveillance system records that could more precisely estimate peak timing.

#### Four seasons

Studies often defined four seasons with equal 3-month lengths and were described as: ‘one of the 4 quarters into which the year is commonly divided’ [38]. Studies consistently named seasons ‘summer’, ‘fall’, ‘winter’ and ‘spring’ despite seasons' start and end dates differing by geographic locations and climatic zones (Supplementary Table S3). Nearly all studies used monthly surveillance data, which inferred that seasons spanned from the first day of the first month to the last day of the third month (CID 17, 36). Studies using daily or weekly surveillance data occasionally selected dates to start and end mid-month (CID 90). Studies used unequal season lengths when analytically defining a high-incidence season (commonly referred to as summer) of 4 rather than 3 months (CID 91–95). Few studies defined high-incidence seasons using environmental factors such as rainfall, temperature or their combination (CID 18, 77, 96).

Quarterly seasons are historically defined by the photoperiod and align with agricultural production schedules [43]. Seasons vary by the relative durations of daylight and depend on a location's position relative to the equator [43]. Since meteorological patterns in each season vary by geographic location, so does the influence of environmental drivers on the peak timing of gastrointestinal infections. As expected, we found that studies conducted in the Southern Hemisphere defined summer/highest-incidence seasons from December to March while studies in the Northern Hemisphere defined summer/highest-incidence seasons from April to September (CID 16, 97–100).

Definitions of seasons also varied within the same geographic location. For example, we identified 10 studies conducted using the FoodNet surveillance system in the United States in 1996–2013 (CID 91, 101–109). Seven studies described seasonal peaks in summer months defined as June to August (CID 101–107). Two studies defined summertime peaks from July to September while one study used a broader 4-month interval from June to September (CID 91, 108, 109). Variation in definitions of summer results in less precise peak timing estimates; when comparing these studies, peak timing ranges from June to September despite most studies having a 3-month season length.

The use of four seasons follows similar analytical advantages and limitations as two seasons. The fixed number of seasons allows researchers to detect seasonality with straightforward statistical analyses that adjust for multiple comparisons. Researchers can incorporate indicator variables in regression models that allow adjusting for additional confounding variables. Differences in season lengths require special attention: researchers must weight differences in the duration of each season. Studies rarely addressed this issue.

**Fig. 3.**
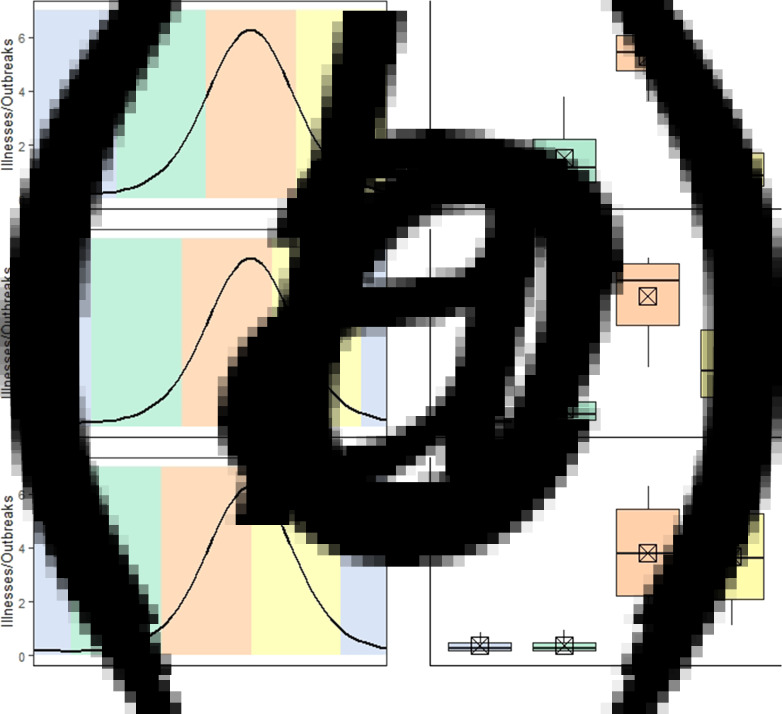
An illustration of detecting seasonality and estimating seasonal peak timing using four discrete seasons. Scenarios include (a) when peak timing is well aligned with the centre of a season; (b) when peak timing is shifted from the centre of an *a priori* assigned season; and (c) when peak timing aligns with the boundary between 2 seasons. Scenario (a) offers higher precision and accuracy as compared to scenarios (b) and (c).

The most accurate detection of seasonality occurs when the seasonal peak falls at the centre of an *a priori* assigned season (Fig. 3a). Researchers' ability to detect seasonality reduces with any misalignment between assigned seasons and the actual seasonal curve (Figs 3b and 3c). When the seasonal peak falls near or at the boundary of two seasons, these seasons are indistinguishable. Researchers may then conclude that an infection peaks within a ~6-month period – a substantially reduced precision compared to quarterly seasons. When discrete seasons are defined by exogenous factors, such as ambient temperature or rainfall, misalignments could indicate a lag between infection and exposure (CID 65).

Defining seasonality with four seasons coarsely aggregates time series data, resulting in less precise estimates than daily, weekly or monthly data could allow for. Data aggregation impedes researchers from examining complex temporal behaviours of seasonal curves to inspecting the reliability of peak timing estimates. Quarterly seasons also lack a uniform environmental or biological factor shared across geographic locations. Comparisons between studies are challenging to assess and may cause confusion given seasons' common names worldwide. Even studies in the same location using the same dataset and study period used dissimilar definitions, reducing the precision of peak timing estimates when comparing these studies.

The four-season method somewhat ignores biologically plausible assumptions of temporality. Take the example of comparing springtime incidence to other seasons. The conceptual interpretation of a difference with a preceding season like winter differs from a comparison to an immediately following season like summer. Furthermore, comparisons between spring and fall fail to account for variations in incidence during summer. The use of discrete seasons overlooks the autoregressive nature of time series data and might draw comparisons based on unrealistic assumptions of temporality.

#### Calendar months

Studies most often used discrete Gregorian calendar months to detect seasonality and estimate peak timing. Researchers applied various statistical methods to monthly time series data (CID 56, 110–113) and estimated peak timing by identifying the calendar month with the highest average incidence or cumulative infections compared to other months (CID 13, 20, 25, 44, 51, 114–118). Many studies with only 1–3 years of surveillance data warranted this method, as more rigorous inspection of seasonal curves was not possible (Supplementary Table S4; CID 119–128).

Few studies with longer study periods conducted Mann–Whitney rank-sum tests with Bonferroni multiple comparison corrections (CID 129–130). Most studies examined seasonality using a discrete, 12-level categorical variable within logistic regression models. Researchers assessed significant differences using OR and IRR measures of association, which compared average incidence in each calendar month to a reference month (CID 34, 56, 110–113). When reference months had lowest incidence, OR and IRR estimates for the highest incidence month were analogous with the seasonal amplitude.

The international recognition of Gregorian calendar months establishes a common terminology for comparing study results. Many surveillance systems publicly report monthly records, allowing researchers to define seasonality by month and coarser seasons as desired (CID 127, 130). Monthly records are not bound to infection incidence or environmental drivers, easing researchers' examination of shifts in peak timing [13]. However, this discrete method still violates assumptions of temporality and ignores the autoregressive nature of time series data. Furthermore, the Gregorian calendar has month length irregularities [14] and often poorly reflects true climatic, social or biological seasons.

Monthly maximums accurately and precisely detect seasonality and estimate peak timing when infections have one consistent annual peak. However, statistical maximums are susceptible to outliers in the distribution of time series data. As noted earlier, many gastrointestinal infections exhibit irregularities in their seasonal pattern when long periods of low incidence alternate with short bursts of infections. This variability can result in numerous months with high incidence or the appearance of bimodal peaks due to outbreak events. While researchers can likely detect seasonality even with these irregularities, the accuracy and precision of peak timing estimates may reduce dramatically.

Few studies conducted formal comparison tests to determine differences between months (Supplementary Table S4). Yet, many studies used inferential language when reporting summaries of monthly incidence that implied statistical significance. Additionally, few studies conducting regression analyses stated the reference month when reporting results. These omissions inhibit reproducibility of analyses and prevent comparisons of study findings.

### Describing seasonality using seasonal curves

Studies more precisely detected seasonality and estimated peak timing when constructing seasonal curves. These methods retained the temporal order of time series data and assumed that incidence at one time point depended on its intensity and variability in preceding time points, also known as autocorrelation (CID 66, 73, 75, 131, 132) [44, 45]. Descriptive studies constructed seasonal curves using various smoothers, spline functions or STL (CID 55, 69, 70, 72, 133–136). SARIMA models, harmonic models and spectral analyses estimated peak timing while adjusting for the periodicity and autoregressive nature of seasonal curves (CID 54, 74, 75, 137–141). Researchers estimated seasonality features and their uncertainty using regression coefficients from fitted models and applications of the *δ*-methods (CID 49, 50, 53, 61–65, 142, 143). The accuracy and precision of peak timing depended on time series length, white noise and patterns of missing data.

#### Splines, smoothers and STL

These methods detected seasonality using moving average smoothing and cubic spline modelling techniques (Supplementary Table S5). Average smoothing used a series of averages estimated on sequential time intervals to remove noise from data (CID 55, 136). Researchers specified the length of time intervals referred to as smoothing windows. Broader windows resulted in more filtering, optimal for examining linear trends while narrower windows resulted in less filtering to reveal seasonal patterns. Researchers detected and estimated seasonality by visual inspection of fitted regression values (CID 55, 136).

Models with embedded spline functions filtered white noise by fitting polynomial functions for sequential time intervals of seasonal curves. Researchers determined interval length by defining breakpoints throughout the curve, referred to as knots (CID 69, 70, 72, 133). Higher order polynomials with more knots had greater noise-to-signal ratios and captured more non-linear fluctuations in incidence like seasonality. Lower order polynomials with fewer knots filtered more white noise and created highly smoothed fitted values approaching linear trends. Researchers determined the presence of seasonality using visual inspection (CID 69, 70, 72, 133). Cubic splines captured seasonal peak and nadir timing more effectively than first- or second-degree polynomial functions.

STL attempted to isolate seasonal trends by decomposing data into three components: (i) a trend model of annual fluctuations; (ii) a seasonal model to account for daily, weekly or monthly fluctuations; and (iii) a model describing residual variability (CID 134, 135) [14]. This partitioning allowed researchers to inspect seasonal patterns using sinusoidal and cosinusoidal harmonic terms while filtering out other components (CID 134, 135). Researchers used rank-based Non-parametric Seasonality Tests (NPST) to estimate peak timing [46].

These methods all filter white noise and irregularities in time series data to reveal trends and seasonality features more clearly. Researchers do not need to aggregate daily and weekly records to precisely describe temporal fluctuations, though daily records may have day-of-the-week effects requiring additional attention. These methods retain the temporal order of data, capture the autocorrelative nature of seasonal curves and estimate peak timing using standardised time units comparable across studies. Researchers can also construct smoothers and splines or perform STL as exploratory data analyses to better specify main regression models.

Application of these techniques depends on how researchers select model parameters and visually inspect smoothed or fitted value curves. Researchers objectively define model parameters, decide window size or knot placement, and select the type of smoothed averaging, degree of polynomial order or method for inspecting seasonal components. These decisions influence the signal-to-noise ratio captured by smoothed or fitted values and necessitate researchers to conduct sensitivity analyses to ensure optimal selection of model parameters.

#### Seasonal ARIMA models, harmonic models and spectral analyses

These methods examined the periodicity of infection seasonality using the concept of harmonic functions in so-called time and frequency domains [47] (Supplementary Table S5). Time domain methods assume a constant periodicity of health outcomes, often annual cycles and include SARIMA models, Fourier series transformations and harmonic regression models. Studies modelled the seasonal curve of infections using a frequency equal to the number of time units per annual cycle (e.g. 365.25 days, 52.25 weeks or 12 months). Researchers detected seasonality based on the significance of sinusoidal and cosinusoidal regression coefficients (CID 35, 38, 54, 68, 144). Studies using SARIMA models often examined associations between the peak timing of infections and environmental risk factors (CID 57, 66, 73, 75). SARIMA models also adjusted for seasonal autocorrelation, or when infection seasonality depends on its periodic behaviour in previous annual cycles (CID 66, 73, 75, 131, 132). Seasonal autocorrelation emphasised the importance of longer time series lengths and fewer missing records [47].

Studies using harmonic regression models adjusted for Poisson or negative binomial distributions due to the non-negative right skewed behaviour of disease incidence (CID 43, 53, 70, 71, 143, 145). All studies assumed that annual infection seasonality aligned with Gregorian calendar years. Few studies estimated seasonal characteristics and their uncertainty measures by applying the *δ*-methods to regression coefficients (CID 49, 50, 53, 61, 62, 65, 76, 142, 143, 146). This technique converted trigonometric regression coefficients from a radial to a linear coordinate system to calibrate and estimate peak timing and amplitude [15]. The *δ*-methods provided both point estimates and confidence intervals for seasonality feature, enabling formal comparisons of estimates across geographic locations, study populations and pathogens (CID 49, 50, 53, 65, 142, 146).

Frequency domain methods do not assume a constant annual cycle and instead examine all possible cycle lengths in a study period (CID 140, 141). Spectral analyses, sometimes referred to as wavelet analyses, estimate peak timing by identifying the cycle length with best model fit (CID 140, 141). This technique was often used in exploratory analyses to determine the optimal cycle length before constructing harmonic regression models (CID 63, 140). Researchers also used spectral analyses to compare the frequency of seasonal gastrointestinal infections with environmental risk factors (CID 141).

Both time and frequency domain methods retain the temporal order, autocorrelative nature and temporal resolution of time series data. Studies reporting peak timing estimates used standardised Gregorian time units, easing comparisons between studies. Trigonometric functions have well-understood harmonic properties that allow for elegant description and estimation of the shape and frequency of seasonal curves. Researchers detected seasonality by the significance of harmonic terms within models and could investigate complex dual peak behaviours or shifts in peak timing by including multiple harmonic terms of varying frequencies.

Time series length and patterns of missing records influence the accuracy and precision of estimating peak timing with these methods. Shorter time series may have case influential observations that distort seasonal patterns of infections. However, researchers using longer time series should adjust for shifts in seasonal peak timing due to surveillance system maturity or changes in environmental drivers of infection over time. The percentage and location of missing data can also distort seasonal curves and bias estimates of seasonality features. Few studies summarised time series length or patterns of missing data.

### Recommendations for improving time series reporting

We summarise the advantages and limitations of each time series method discussed above (Table 2). We hope this overview provides general guidelines to inform decision-making processes of researchers for selecting and utilizing time series methods. We encourage academic journal reviewers to use these guidelines when commenting on the appropriateness and completeness of time series methods in submitted manuscripts. Our overview demonstrates that, while discrete methods are computationally straightforward and require minimal statistical training, the use of seasonal curves provide more reliable description of seasonal patterns and estimation of seasonality features.

**Table 2.** Overview of the advantages (✓) and limitations (✗) of time series methods described in this systematic review

	2 and 4 Seasons	Calendar months	Smoothers, splines, STL	SARIMA, harmonics	Spectral analyses	*δ*-Methods
Minimal statistical training is required to calculate peak timing estimates	✓	✓	✗	✗	✗	✗
Definitions of seasons and peak timing estimates are easily comprehended by general audiences	✓	✓	✗	✗	✗	✗
Researchers do not need to aggregate surveillance data to perform time series analyses	✗	✓	✓	✓	✓	✓
Statistical results are generalisable using standardised Gregorian calendar time units	✗	✓	✓	✓	✓	✓
Researchers do not define seasons' lengths or reference seasons (i.e. data-driven analyses)	✗	✗	✓	✓	✓	✓
Time series methods adjust for irregularities and temporality of surveillance data	✗	✗	✓	✓	✓	✓
Time series methods do not require long time series to achieve sufficient sample size	✗	✗	✓	✓	✓	✓
Modelling techniques are flexible to adjust for other time-varying risk factors	✗	✗	✓	✓	✓	✓
Modelling techniques are flexible to adjust for dual peak or multi-peak outbreak behaviours	✗	✗	✗	✓	✓	✓
Modelling techniques adjust for harmonic seasonal curves using trigonometric functions	✗	✗	✗	✓	✓	✓
Modelling techniques are reliable at detecting seasonality using regression coefficients	✗	✗	✗	✓	✓	✓
Modelling techniques offer robust peak estimate in the presence of multiple seasonal peaks	✗	✗	✗	✗	✓	✓
Modelling techniques calculate seasonality features with measures of uncertainty	✗	✗	✗	✗	✗	✓
Peak timing estimates can be easily compared across subpopulations, pathogens, locations, etc.	✗	✗	✗	✗	✗	✓

Methods detect seasonality and estimate peak timing using discrete comparisons between seasons (two seasons or four seasons; calendar months) and constructed seasonal curves (smoothing, spline and STL methods; SARIMA, Fourier series transformations and harmonic models without applying the *δ*-methods; harmonic models with applying the *δ*-methods; and spectral analyses). We differentiate the advantages and limitations of harmonic models that do and do not apply the *δ*-methods to emphasise the importance of these methods in infectious disease seasonality research.

In addition, we propose a glossary of terms to standardise the reporting of results when using time series analyses (Table 3). As noted above, researchers used terms like *trends*, *seasonal trends* and *seasonal peaks* interchangeably, yet their meaning and interpretation vary dramatically. While results inferred statistical significance, studies often neither conducted nor reported comparison test results. Non-standardised terminology also yields uncertainty of statistical methods, misleading study findings, and potentially inaccurate estimations of peak timing. Terms defined in Table 3 can provide clearer aims, objectives and outcomes of studies using time series methods to describe, compare, explain and predict infection seasonality. Standardised terminology must accompany improved clarity in the reporting of case definitions and date of illness onset, testing and laboratory confirmation, as these differences may lead to unreliable comparisons of seasonality features across surveillance systems. Establishing consensus also invites interdisciplinary collaboration and sharing of methods for modelling surveillance data.

**Table 3.** A summary of terminology for describing time series analyses conducted in infectious disease epidemiology research

Term	Definition
Time series data	A set or a sample of time-referenced observations or records with an identified time period, cycle and unit (e.g. day, week, month extracted from a timestamp as YYYY:MM:DD:HH:mm) often illustrated by dot, line or needle plots with axes reflecting time and an outcome of interest.
Distribution of time series data	A general summary of frequencies in time-referenced data – i.e. how often an outcome of interest reaches a certain level with respect to time units (often illustrated with histograms and density plots).
Time series analyses	A collection of methods to describe, explain and predict temporal processes with time-referenced data for an outcome of interest.
Trend	General temporal behaviour in an outcome of interest that can exhibit steady incremental changes (linear) or varying incremental changes (non-linear) over time.
Season	An interval of time within one time cycle (typically 1 calendar year) defined by a specific biological, environmental, physical, physiological, or other property or feature in a biological or non-biological system [24].
Seasonal pattern	A recurrence of periods in an outcome of interest with alternating values (e.g. high and low) over the course of a time cycle.
Seasonality	A systematic periodic fluctuation in an outcome of interest over the course of one cycle (typically 1 calendar year) as an observable property of a biological or non-biological system.
Seasonal curve	An analytical representation of seasonal periodic fluctuations in an outcome of interest within one time cycle (typically 1 calendar year).
Seasonality features	A set of measurable characteristics to describe seasonality and a seasonal curve within 1 year, including seasonal peak, nadir, intensity, duration, speed at which a seasonal curve reaches its peak and speed at which a seasonal curve declines to its nadir [17].
Peak or nadir timing	A seasonality feature that represents times when a seasonal curve of an outcome reaches its maximum or minimum [17].
Amplitude or intensity	A seasonality feature that represents the difference between seasonal peaks and nadirs [17].
Duration	A seasonality feature that represents the time interval when incidence rises above a specified threshold [17].

Terms specify differences between time series data, distributions and analyses, as well as trends, season, seasonal patterns, seasonality and seasonality features.

## Discussion

In this review, we described time series methods using discrete seasons and seasonal curves to investigate the seasonality of gastrointestinal infections. Discrete seasons are prone to misclassification biases and studies lacked standardised terminology for reporting results and formal comparison tests. Studies constructing seasonal curves used methods that accounted for the temporality and autocorrelation of data. However, these studies often failed to estimate peak timing or thoroughly report on data limitations. We recommend that researchers: (i) use standardised terminology when reporting seasonality features; (ii) utilise more rigorous methods when estimating peak timing; and (iii) provide greater attention to data limitations when applying time series methods.

Our review revealed that a study's selection of time series methods relates closely to its underlying assumptions and rationale. Descriptive studies often estimate peak timing as the season or calendar month of maximal incidence while comparative or explanatory studies use seasonal curves to examine associations between illnesses and their drivers. However, we noted the stringency of selected methods depended not on study intent but rather the availability of usable data, dictating the acquired precision of seasonality estimates. The assumptions are rarely specified, yet they are essential for proper selection of both study design and methods of data analysis. These assumptions refer to researchers' ability to ensure optimal analytic power to determine the relevant mechanisms governing human infections. The first assumption is that a selected seasonal curve (or model) is optimal for describing the observable temporal processes; the ability to meet this assumption dictates the approach of handling noise due to poor fit or outliers when observed data are not aligned or do not lie sufficiently close to a selected curve. An additional assumption is that the selected populations are optimal for both observing and generating a seasonal curve (or model) for a selected pathogen. To test whether a study can meet these assumptions, we need rich and solid methodology development aiming to further improve research quality.

Studies often failed to report the lengths and dates of discrete seasons or to state reference seasons or months when reporting results. These omissions reduce the reliability of peak timing estimates, inhibit the reproducibility of analyses and prevent comparisons of study findings. Researchers must comprehensively report season lengths and dates given the variability of environmentally- or culturally-derived seasons by climate zone and geographic location. This is especially pertinent when using four seasons, which lack uniform environmental or biological factors shared across geographic locations.

While we support defining seasons by solstices and equinoxes due their uniformity, we also recognise the natural changes of the photoperiod during those intervals across latitudes [48]. Photoperiod intervals may fail to capture the relevant environmental drivers of disease transmission in all locations. Discrete seasons may also fail to capture seasonal patterns caused by non-seasonal drivers such as extreme weather events, livestock migration or practices around food, water and hygiene [20–22]. The complexity of defining seasons dictates the need for hypothesis-generating exploratory analyses to better choose a seasonal form and a detailed plan for sensitivity analyses assessing these alternatives. Furthermore, detecting the changes in seasonal features are valuable indicators of how circulating strains [30, 49–52], extreme events [53] or seasonal host migration [54] could alter seasonal behaviours. When seasonal peaks differ across subpopulations, it could indicate different underlying environmental mechanisms and interactions and subsequent disease transmission to humans. To detect such differences, granular temporal, spatial and etiological data with capabilities for global standardisation and harmonisation are needed.

High-resolution data maximise the potential of surveillance systems for describing, comparing and explaining changes in disease incidence over time and across species, pathogens, locations and other dimensions. Worldwide, public health agencies routinely collect time-referenced records to monitor the incidence of gastrointestinal infections and outbreaks. Surveillance systems track many common pathogens, such as *Salmonella*, *Campylobacter*, *Listeria* and *E. coli*, which contribute to ~600 million infections worldwide annually [9, 30–33]. Indicator-based surveillance systems (structured data with formal case definitions) reflect the total number of persons infected per unit of time [1, 13]. Event-based surveillance systems (public health emergencies reported using unstructured information) record implicated food and water sources linked to disease outbreaks [1]. The growing accuracy and precision of surveillance data could offer new opportunities for timely detection of changes in seasonal patterns.

The rapid adoption of growing analytical tools helps researchers to better model disease seasonality. Better understanding of how to construct seasonal curves will encourage disease surveillance systems to collect and report data with the highest temporal and spatial resolution possible. Broader utilisation of the modern time series models is needed to improve precision of peak timing estimates. Approaches like the *δ*-methods simultaneously detect the presence of seasonality and quantify seasonality features [13, 15, 28]. Most importantly, the *δ*-methods provide confidence intervals, allowing for formal statistical comparisons by location, subpopulation, pathogen, etc. [12, 16, 17].

Our review revealed that researchers underreport data limitations when applying time series methods. Maximum average incidence or cumulative infections can be biased by case influential observations or shifts in peak timing masked by coarse data aggregation. Studies constructing seasonal curves face data limitations like time series length and patterns of missing records. Time series length influences the effective sample size for regression analyses while the quantity and location of missing records influence the precision of peak timing estimates. Techniques for investigating the patterns of missing records in time series data have been explored for influenza [55]. We recommend that public health agencies and researchers report similar metrics as metadata to accompany the publicly available surveillance data they report and use, respectively.

Reliable estimation of peak timing permits targeted and proactive public health interventions to dampen seasonal infection incidence and prevent widespread outbreaks [12]. Public health agencies have expanded surveillance coverage in recent decades using collaborative networks of testing laboratories and public health agencies [56, 57]. More rigorous investigation of available data can improve: (i) early warning forecast accuracy to reduce socio-economic burdens of disease; (ii) fiscal and personnel resource allocations in medical facilities to treat higher seasonal patient volumes; (iii) laboratory testing supplies management; and (iv) the timeliness and effectiveness of food and water safety inspections [34, 35, 58, 59]. After the influenza pandemic of 2009, we observed an unprecedented growth in testing and reporting capacities worldwide [55]. The global response to the ongoing pandemic of COVID-19 has demonstrated further improvements in tracking infections. These newly acquired skills and partnerships should now be directed toward better monitoring of seasonal infections causing extensive health and economic burdens.

## Conclusion

Greater attention to how seasonality is defined, modelled, interpreted and reported is necessary to promote reproducible research. Our review shows that applications of advanced time series analyses are underutilised in epidemiological research on enteric infections. We encourage increased training in these methods to incite more rigorous assessment of disease seasonality. These methods can provide more reliable estimation of infection peak timing while properly adjusting for irregularities of infections' temporal occurrences. Standardised terminology and more informative reporting requirements on data limitations will promote interdisciplinary collaborations in the modelling and forecasting of infectious diseases. A deeper understanding of the utility of time series analyses will encourage more expansive collection of surveillance data and refined temporal and spatial resolution when reporting that data.

## Data Availability

The data that support the findings of this study are openly available in Supplementary Table S1.

## Supplementary material

For supplementary material accompanying this paper visit http://doi.org/10.1017/S0950268822000243. click here to view supplementary material
