# Supplementary material for: Investigating seasonal patterns in enteric infections: a systematic review of time series methods
Source: Epidemiol Infect. 2022 Feb 14;150:e50. doi: 10.1017/S0950268822000243 (PMC8915194; doi:10.1017/S0950268822000243)
Supplement: Supplementary file 1 [file S0950268822000243sup001.docx]

**Epidemiology and Infection**

Supplemental Materials

Investigating seasonal patterns in enteric infections: a systematic review of time series methods

**Ryan B. Simpson^1^, Alexandra V. Kulinkina^1,2,3^, Elena N. Naumova^1,^***

^1^ Tufts University Friedman School of Nutrition Science and Policy, Boston, MA, USA 02111; [ryan.simpson@tufts.edu](mailto:ryan.simpson@tufts.edu); [elena.naumova@tufts.edu](mailto:elena.naumova@tufts.edu); [alexandra.kulinkina@swisstph.ch](mailto:alexandra.kulinkina@swisstph.ch)

^2^  Swiss Tropical and Public Health Institute, Basel, Switzerland; [alexandra.kulinkina@swisstph.ch](mailto:alexandra.kulinkina@tufts.edu)

^3^  University of Basel, Basel, Switzerland; [alexandra.kulinkina@swisstph.ch](mailto:alexandra.kulinkina@tufts.edu)

**Corresponding Author:**

Elena N. Naumova

[elena.naumova@tufts.edu](mailto:elena.naumova@tufts.edu)

150 Harrison Avenue, Boston, MA 02111

**Word Count:** 6312 words

Epidemiology and Infection

*Supplemental Materials*

Investigating seasonal patterns in enteric infections: a systematic review of time series methods

**Supplemental Table S1.** A list of all citations (n=220) included in our systematic literature review outlining advantages and limitations of common methods for estimating seasonal peak timing. Studies were original research articles that detected and estimated the seasonality of human gastrointestinal infections using local, regional, and national surveillance systems or hospital health records. We provide a citation identifier (CID) and the full citation for each study that is referred to within the manuscript. Studies are listed in the order of their appearance within the manuscript and then alphabetically for those not referenced.

| **CID** | **Citation** |
| --- | --- |
| ***Citations Referenced in Manuscript Text (in order of appearance)*** | |
| 1 | **Li Y, *et al***. (2014) Nontyphoidal *Salmonella* infection in children with acute gastroenteritis: prevalence, serotypes, and antimicrobial resistance in Shanghai, China. *Foodborne Pathogens and Disease*; **11**: 200-206. |
| 2 | **Ran L, *et al***. (2011) Laboratory-based surveillance of nontyphoidal *Salmonella* infections in China. *Foodborne Pathogens and Disease*; **8**: 921-927. |
| 3 | **Hendriksen RS, *et al***. (2009) Risk factors and epidemiology of the ten most common *Salmonella* serovars from patients in Thailand: 2002-2007. *Foodborne Pathogens and Disease*; **6**: 1009-1019. |
| 4 | **Iritani N, *et al***. (2019) GII.17 norovirus infections in outbreaks of acute nonbacterial gastroenteritis in Osaka City, Japan during two decades. *Journal of Medical Virology*; **91**: 2101-2107. |
| 5 | **Mukherjee S, *et al***. (2019) Increasing frequencies of antibiotic resistant non-typhoidal *Salmonella* infections in Michigan and risk factors for disease. *Frontiers in Medicine*; **6**: 250. |
| 6 | **Oloya J, *et al***. (2007) Evaluation of *Salmonella* occurrence in domestic animals and humans in North Dakota (2000-2005). *Foodborne Pathogens and Disease*; **4**: 551-563. |
| 7 | **Mason J, *et al***. (2013) *Campylobacter* infection in children in Malawi is common and is frequently associated with enteric virus co-infections. *PLOS One*; **8**: e59663. |
| 8 | **Lian Y, *et al***. (2019) Epidemiology of norovirus outbreaks reported to the Public Health Emergency Event Surveillance System, China, 2014⁻2017. *Viruses*; **11**: 342. |
| 9 | **Yoder JS, Beach MJ**. (2007) Giardiasis surveillance--United States, 2003-2005. *Morbidity and Mortality Weekly Report*; **56**: 11-18. |
| 10 | **Yoder JS, Harral C, Beach MJ**. (2010) Giardiasis surveillance - United States, 2006-2008. *Morbidity and Mortality Weekly Report*; **59**: 15-25. |
| 11 | **Yoder JS, *et al***. (2012) Giardiasis surveillance--United States, 2009-2010. *Morbidity and Mortality Weekly Report*; **61**: 13-23. |
| 12 | **Collard JM, *et al***. (2008) Drastic decrease of *Salmonella enteritidis* isolated from humans in Belgium in 2005, shift in phage types and influence on foodborne outbreaks. *Epidemiology and Infection*; **136**: 771-781. |
| 13 | **Bennett SD, *et al***. (2018) Produce-associated foodborne disease outbreaks, USA, 1998-2013. *Epidemiology and Infection*; **146**: 1397-1406. |
| 14 | **Guzman-Herrador B, *et al*.** (2015) Waterborne outbreaks in the Nordic countries, 1998 to 2012. *Eurosurveillance*; **20**: 21160. |
| 15 | **Rush BA, Chapman PA, Ineson RW**. (1990) A probable waterborne outbreak of cryptosporidiosis in the Sheffield area. *Journal of Medical Microbiology*; **32**: 239-242. |
| 16 | **Yun YS, *et al***. (2021) The prevalence and characteristics of shiga toxin-producing *Escherichia coli* isolated by the Enteric Pathogens Active Surveillance Network (Enter-Net) in the Republic of Korea, 2009-2018. *Microbial Pathogenesis*; **158**: 105005. |
| 17 | **Sher AA, *et al***. (2021) Epidemiological trends of foodborne *Campylobacter* outbreaks in the United States of America, 1998-2016. *Food Microbiology*; **97**: 103751. |
| 18 | **Kim JG, Kim JS, Kim JG.** (2021) Characteristics of norovirus food poisoning outbreaks in Korea in the 2000s. *Journal of Food Protection;* **84**: 472-480. |
| 19 | **Al Dahouk S, *et al***. (2007) Changing epidemiology of human brucellosis, Germany, 1962-2005. *Emerging Infectious Diseases*; **13**: 1895-1900. |
| 20 | **Craun GF**. (1979) Waterborne giardiasis in the United States: a review. *American Journal of Public Health*; **69**: 817-819. |
| 21 | **Olsen SJ, *et al***. (2001) The changing epidemiology of *Salmonella*: trends in serotypes isolated from humans in the United States, 1987-1997. *The Journal of Infectious Diseases;* **183**: 753-761. |
| 22 | **Chui KK, *et al****.* (2009) Geographic variations and temporal trends of *Salmonella*-associated hospitalization in the U.S. elderly, 1991-2004: a time series analysis of the impact of HACCP regulation. *BMC Public Health*; **9**: 447. |
| 23 | **Barker WH Jr., *et al***. (1974) Foodborne disease surveillance: Washington State. *American Journal of Public Health*; **64**: 854-859. |
| 24 | **Fodor T, *et al***. (1970) Food poisoning occurrences in New York City, 1969. *Public Health Reports*; **85**: 1013-1018. |
| 25 | **Dietz V, *et al***. (2000) Active, multisite, laboratory-based surveillance for *Cryptosporidium parvum*. *The American Journal of Tropical Medicine and Hygiene;* **62**: 368-372. |
| 26 | **Grass JE, Gould LH, Mahon BE**. (2013) Epidemiology of foodborne disease outbreaks caused by *Clostridium perfringens*, United States, 1998-2010. *Foodborne Pathogens and Disease*; **10**: 131-136. |
| 27 | **Hall RL, *et al***. (2012) Population-based active surveillance for *Cyclospora* infection--United States, Foodborne Diseases Active Surveillance Network (FoodNet), 1997-2009. *Clinical Infectious Diseases*; **54** (Suppl. 5): S411-417. |
| 28 | **Lopman BA, *et al***. (2003) Two epidemiologic patterns of norovirus outbreaks: surveillance in England and Wales, 1992-2000. *Emerging Infectious Diseases*; **9**: 71-77. |
| 29 | **Lee WC, *et al***. (2001) Foodborne illness outbreaks in Korea and Japan studied retrospectively. *Journal of Food Protection;* **64**: 899-902. |
| 30 | **Casillas SM, Hall RL, Herwaldt BL**. (2019) Cyclosporiasis surveillance - United States, 2011-2015. *Morbidity and Mortality Weekly Report*; **68**: 1-16. |
| 31 | **Michel P, *et al***. (2006) Regional, seasonal, and antimicrobial resistance distributions of *Salmonella typhimurium* in Canada: a multi-provincial study. *Canadian Journal of Public Health*; **97**: 470-474. |
| 32 | **Ford MW, *et al***. (2003) A descriptive study of human *Salmonella* serotype typhimurium infections reported in Ontario from 1990 to 1998. *Canadian Journal of Infectious Diseases and Medical Microbiology;* **14**: 267-273. |
| 33 | **Younus M, *et al***. (2006) Demographic risk factors and incidence of *Salmonella enteritidis* infection in Michigan. *Foodborne Pathogens and Disease*; **3**: 266-273. |
| 34 | **Ravel A, *et al***. (2010) Seasonality in human salmonellosis: assessment of human activities and chicken contamination as driving factors. *Foodborne Pathogens and Disease*; **7**: 785-794. |
| 35 | **David JM, *et al***. (2017) Do contamination of and exposure to chicken meat and water drive the temporal dynamics of campylobacter cases? *Epidemiology and Infection*; **145**: 3191-3203. |
| 36 | **Decuir M, *et al***. (2021) Evidence of false positivity for *Vibrio* species tested by gastrointestinal multiplex PCR panels, Minnesota, 2016-2018. *Open Forum Infectious Diseases*; **8**: ofab247. |
| 37 | **Martinez PP, *et al***. (2020) Tube well use as protection against rotavirus infection during the monsoons in an urban setting. *The Journal of Infectious Diseases;* **221**: 238-242. |
| 38 | **Rushton SP, *et al***. (2019) Climate, human behaviour or environment: individual-based modelling of *Campylobacter* seasonality and strategies to reduce disease burden. *Journal of Translational Medicine*; **17**: 34. |
| 39 | **Blanton LH, *et al***. (2006) Molecular and epidemiologic trends of caliciviruses associated with outbreaks of acute gastroenteritis in the United States, 2000-2004. *The Journal of Infectious Diseases;* **193**: 413-421. |
| 40 | **Kelly S, *et al***. (2008) Establishment of a national database to link epidemiological and molecular data from norovirus outbreaks in Ireland. *Epidemiology and Infection*; **136**: 1472-1479. |
| 41 | **Kroneman A, *et al***. (2008) Analysis of integrated virological and epidemiological reports of norovirus outbreaks collected within the Foodborne Viruses in Europe network from 1 July 2001 to 30 June 2006. *Journal of Clinical Microbiology*; **46**: 2959-2965. |
| 42 | **Arshad MM, *et al***. (2007) A registry-based study on the association between human salmonellosis and routinely collected parameters in Michigan, 1995-2001. *Foodborne Pathogens and Disease*; **4**: 16-25. |
| 43 | **Li X, *et al***. (2020) Spatial epidemiology of salmonellosis in Florida, 2009-2018. *Frontiers in Public Health*; **8**: 603005. |
| 44 | **Bennion JR, *et al***. (2008) Decreasing listeriosis mortality in the United States, 1990-2005. *Clinical Infectious Diseases;* **47**: 867-874. |
| 45 | **Dmochowska P, *et al***. (2019) Epidemiological survey and retrospective analysis of salmonella infections between 2000 and 2017 in Warmia and Masuria Voivodship in Poland. *Medicina*; **55**: 74. |
| 46 | **Kuhn KG, *et al***. (2018) Epidemiology of campylobacteriosis in Denmark 2000-2015. *Zoonoses and Public Health*; **65**: 59-66. |
| 47 | **Samuel MC, *et al***. (2004) Epidemiology of sporadic *Campylobacter* infection in the United States and declining trend in incidence, FoodNet 1996-1999. *Clinical Infectious Diseases;* **38** (Suppl. 3): S165-S174. |
| 48 | **Emch M, *et al***. (2008) Seasonality of cholera from 1974 to 2005: a review of global patterns. *International Journal of Health Geographics*; **7**: 1-13. |
| 49 | **Chui KK, *et al***. (2011) Visual analytics for epidemiologists: understanding the interactions between age, time, and disease with multi-panel graphs. *PLOS One*; **6**: e14683. |
| 50 | **Chui KK, *et al***. (2011) Hospitalization of the elderly in the United States for nonspecific gastrointestinal diseases: a search for etiological clues. *American Journal of Public Health*; **101**: 2082-2086. |
| 51 | **Painter JE, *et al***. (2015) Giardiasis surveillance -- United States, 2011-2012. *Morbidity and Mortality Weekly Report*; **64**: 15-25. |
| 52 | **Patrick ME, *et al***. (2014) Incidence of *Cronobacter spp*. infections, United States, 2003-2009. *Emerging Infectious Diseases*; **20**: 1520-1523. |
| 53 | **Simpson RB, Zhou B, Naumova EN**. (2020) Seasonal synchronization of foodborne outbreaks in the United States, 1996-2017. *Scientific Reports*; **10**: 17500. |
| 54 | **White A, *et al***. (2016) Food source prediction of shiga toxin-producing *Escherichia coli* outbreaks using demographic and outbreak characteristics, United States, 1998-2014. *Foodborne Pathogens and Disease*; **13**: 527-534. |
| 55 | **Lake IR, *et al***. (2019) Exploring *Campylobacter* seasonality across Europe using The European Surveillance System (TESSy), 2008 to 2016. *Eurosurveillance*; **24**: 1800028. |
| 56 | **D'Souza RM, *et al***. (2004) Does ambient temperature affect foodborne disease? *Epidemiology*; **15**: 86-92. |
| 57 | **Sebastian T, *et al***. (2015) Role of seasonality and rainfall in *Vibrio cholerae* infections: a time series model for 11 years surveillance data. *Clinical Epidemiology and Global Health*; **3**: 144-148. |
| 58 | **Iyer V, *et al***. (2021) Role of extreme weather events and El Niño Southern Oscillation on incidence of enteric fever in Ahmedabad and Surat, Gujarat, India. *Environmental Research*; **196**: 110417. |
| 59 | **Colston JM, *et al***. (2019) Use of earth observation-derived hydrometeorological variables to model and predict rotavirus infection (MAL-ED): a multisite cohort study. *The Lancet Planetary Health*; **3**: e248-e258. |
| 60 | **Omore R, *et al***. (2016) Epidemiology, seasonality and factors associated with rotavirus infection among children with moderate-to-severe diarrhea in rural Western Kenya, 2008-2012: The Global Enteric Multicenter Study (GEMS). *PLOS One*; **11**: e0160060. |
| 61 | **Kulinkina AV, *et al***. (2016) Seasonality of water quality and diarrheal disease counts in urban and rural settings in south India. *Scientific Reports*; **6**: 20521. |
| 62 | **Stashevsky PS, *et al***. (2019) Agglomerative clustering of enteric infections and weather parameters to identify seasonal outbreaks in cold climates. *International Journal of Environmental Research and Public Health*; **16**: 2083. |
| 63 | **Alsova OK, Loktev VB, Naumova EN**. (2019) Rotavirus seasonality: an application of singular spectrum analysis and polyharmonic modeling. *International Journal of Environmental Research and Public Health*; **16**: 4309. |
| 64 | **Ureña-Castro K, *et al*.** (2019) Seasonality of rotavirus hospitalizations at Costa Rica’s National Children’s Hospital in 2010–2015. *International Journal of Environmental Research and Public Health*; **16**: 2321. |
| 65 | **Naumova EN, *et al***. (2007) Seasonality in six enterically transmitted diseases and ambient temperature. *Epidemiology and Infection*; **135**: 281-292. |
| 66 | **Hsiao HI, Jan MS, Chi HJ**. (2016) Impacts of climatic variability on *Vibrio parahaemolyticus* outbreaks in Taiwan. *International Journal of Environmental Research and Public Health*; **13**: 188. |
| 67 | **Hulland E, *et al***. (2019) Increase in reported cholera cases in Haiti following hurricane Matthew: an interrupted time series model. *The American Journal of Tropical Medicine and Hygiene;* **100**: 368-373. |
| 68 | **Kovats RS, *et al***. (2004) The effect of temperature on food poisoning: a time-series analysis of salmonellosis in ten European countries. *Epidemiology and Infection*; **132**: 443-453. |
| 69 | **Lal A, Konings P**. (2018) Beyond reasonable drought: hotspots reveal a link between the 'Big Dry' and cryptosporidiosis in Australia's Murray Darling Basin. *Journal of Water and Health*; **16**: 1033-1037. |
| 70 | **Leckebusch GC, Abdussalam AF**. (2015) Climate and socioeconomic influences on interannual variability of cholera in Nigeria. *Health and Place*; **34**: 107-117. |
| 71 | **Logar-Henderson C, *et al***. (2019) Effects of large-scale oceanic phenomena on non-cholera vibriosis incidence in the United States: implications for climate change. *Epidemiology and Infection*; **147**: e243. |
| 72 | **Louis VR, *et al***. (2005) Temperature-driven *Campylobacter* seasonality in England and Wales. *Applied and Environmental Microbiology*; **71**: 85-92. |
| 73 | **Park MS, Park KH, Bahk GJ**. (2018) Combined influence of multiple climatic factors on the incidence of bacterial foodborne diseases. *Science of the Total Environment*; **610**: 10-16. |
| 74 | **Rosenberg A, *et al***. (2018) Ambient temperature and age-related notified *Campylobacter* infection in Israel: A 12-year time series study. *Environmental Research*; **164**: 539-545. |
| 75 | **Stephen DM, Barnett AG**. (2016) Effect of temperature and precipitation on salmonellosis cases in South-East Queensland, Australia: an observational study. *BMJ Open*; **6**: e010204. |
| 76 | **White AN, *et al***. (2009) Environmental determinants of campylobacteriosis risk in Philadelphia from 1994 to 2007. *EcoHealth*; **6**: 200-208. |
| 77 | **Campbell AM, *et al***. (2020) Cholera risk: a machine learning approach applied to essential climate variables. *International Journal of Environmental Research and Public Health*; **17**: 9378. |
| 78 | **Chalmers RM, *et al***. (2019) Analysis of the *Cryptosporidium spp*. and gp60 subtypes linked to human outbreaks of cryptosporidiosis in England and Wales, 2009 to 2017. *Parasites and Vectors*; **12**: 95. |
| 79 | **Vega E, *et al***. (2014) Genotypic and epidemiologic trends of norovirus outbreaks in the United States, 2009 to 2013. *Journal of Clinical Microbiology*; **52**: 147-155. |
| 80 | **Tremblay M, *et al***. (2017) Evaluation of the use of zero-augmented regression techniques to model incidence of *Campylobacter* infections in FoodNet. *Foodborne Pathogens and Disease*; **14**: 587-592. |
| 81 | **Yoshikura H**. (2015) Attack rate in food poisoning: order in chaos. *Japanese Journal of Infectious Diseases;* **68**: 394-406. |
| 82 | **Drayna P, *et al***. (2010) Association between rainfall and pediatric emergency department visits for acute gastrointestinal illness. *Environmental Health Perspectives;* **118**: 1439-1443. |
| 83 | **Chhetri BK, *et al***. (2019) Projected local rain events due to climate change and the impacts on waterborne diseases in Vancouver, British Columbia, Canada. *Journal of Environmental Health*; **18**: 116. |
| 84 | **Milazzo A, *et al***. (2016) Heatwaves differentially affect risk of *Salmonella* serotypes. *Journal of Infection*; **73**: 231-240. |
| 85 | **Wu WY, *et al***. (2012) Impact of integrating public health clinical decision support alerts into electronic health records on testing for gastrointestinal illness. *Journal of Public Health Management and Practice*; **18**: 224-227. |
| 86 | **Gillespie IA, *et al***. (2009) Disease presentation in relation to infection foci for non-pregnancy-associated human listeriosis in England and Wales, 2001 to 2007. *Journal of Clinical Microbiology*; **47**: 3301-3307. |
| 87 | **Toyofuku H**. (2008) Epidemiological data on food poisonings in Japan focused on *Salmonella*, 1998-2004. *Food Additives and Contaminants: Part A: Chemistry, Analysis, Control, Exposure and Risk Assessment*; **25**: 1058-1066. |
| 88 | **Carrel M, *et al***. (2009) Spatio-temporal clustering of cholera: the impact of flood control in Matlab, Bangladesh, 1983-2003. *Health and Place*; **15**: 741-752. |
| 89 | **Alexander KA, Heaney AK, Shaman J**. (2018) Hydrometeorology and flood pulse dynamics drive diarrheal disease outbreaks and increase vulnerability to climate change in surface-water-dependent populations: A retrospective analysis. *PLOS Medicine*; **15**: e1002688. |
| 90 | **Judd MC, *et al***. (2019) Epidemiologic patterns of human *Salmonella* serotype diversity in the USA, 1996-2016. *Epidemiology and Infection*; **147**: e187. |
| 91 | **Koehler KM, *et al***. (2006) Population-based incidence of infection with selected bacterial enteric pathogens in children younger than five years of age, 1996-1998. *The Pediatric Infectious Disease Journal*; **25**: 129-134. |
| 92 | **Hill SE, Poss DE, Harris S**. (2017) Incidence of gastrointestinal infections among U.S. active component service members stationed in the U.S. compared to U.S. civilians, 2012-2014. *Medical Surveillance Monthly Report*; **24**: 20-25. |
| 93 | **Yoder JS, Beach MJ**. (2007) Cryptosporidiosis surveillance--United States, 2003-2005. *Morbidity and Mortality Weekly* Report; **56**: 1-10. |
| 94 | **Yoder JS, Beach** **MJ**. (2010) Cryptosporidium surveillance and risk factors in the United States. *Experimental Parasitology*; **124**: 31-39. |
| 95 | **Yoder JS, Harral C, Beach MJ**. (2010) Cryptosporidiosis surveillance - United States, 2006-2008. *Morbidity and Mortality Weekly Report*; **59**: 1-14. |
| 96 | **Flores Monter YM, *et al***. (2021) Edaphoclimatic seasonal trends and variations of the *Salmonella spp*. infection in Northwestern Mexico. *Infectious Disease Modelling*; **6**: 805-819. |
| 97 | **Jeffs E, *et al***. (2019) Epidemiology of *Campylobacter* gastroenteritis in New Zealand children and the effect of the *Campylobacter* strategy: a 20-year observational study. *The Pediatric Infectious Disease Journal*; **38**: 569-576. |
| 98 | **Wagner VR, Silveira JB, Tondo EC**. (2013) Salmonelloses in the State of Rio Grande do Sul, southern Brazil, 2002 to 2004. *Brazilian Journal of Microbiology*; **44**: 723-729. |
| 99 | **Gillespie IA, *et al***. (2003) Milkborne general outbreaks of infectious intestinal disease, England and Wales, 1992-2000. *Epidemiology and Infection*; **130**: 461-468. |
| 100 | **Hu P, *et al*.** (2020) FilmArray GI-panel performance for the rapid and multiple detection of gastrointestinal microorganisms in foodborne illness outbreaks in Shenzhen during 2018-2019. *Journal of Molecular Epidemiology and Evolutionary Genetics of Infectious Diseases*; **86**: 104607. |
| 101 | **Shallow S, *et al***. (1997) Foodborne Diseases Active Surveillance Network, 1996. *Morbidity and Mortality Weekly Report*; **46**: 258-261. |
| 102 | **Shallow S, *et al***. (1998) Incidence of foodborne illnesses--FoodNet, 1997. *Morbidity and Mortality Weekly Report*; **47**: 782-786. |
| 103 | **Shallow S, *et al***. (1999) Incidence of foodborne illnesses: preliminary data from the Foodborne Diseases Active Surveillance Network (FoodNet)--United States, 1998. *Morbidity and Mortality Weekly Report*; **48**: 189-194. |
| 104 | **Wallace DJ, *et al***. (2000) Incidence of foodborne illnesses reported by the Foodborne Diseases Active Surveillance Network (FoodNet)-1997. FoodNet Working Group. *Journal of Food Protection;* **63**: 807-809. |
| 105 | **Ailes E, *et al***. (2008) Continued decline in the incidence of *Campylobacter* infections, FoodNet 1996-2006. *Foodborne Pathogens and Disease*; **5**: 329-337. |
| 106 | **Ong KL, *et al***. (2012) Strategies for surveillance of pediatric hemolytic uremic syndrome: Foodborne Diseases Active Surveillance Network (FoodNet), 2000-2007. *Clinical Infectious Diseases;* **54** (Suppl. 5): S424-S431. |
| 107 | **Ong KL, *et al***. (2012) Changing epidemiology of *Yersinia enterocolitica* infections: markedly decreased rates in young black children, Foodborne Diseases Active Surveillance Network (FoodNet), 1996-2009. *Clinical Infectious Diseases;* **54** (Suppl. 5): S385-S390. |
| 108 | **Crim SM, *et al***. (2018) *Salmonella enterica* Serotype Newport infections in the United States, 2004-2013: increased incidence investigated through four surveillance systems. *Foodborne Pathogens and Disease*; **15**: 612-620. |
| 109 | **Shiferaw B, *et al***. (2004) Trends in population-based active surveillance for shigellosis and demographic variability in FoodNet sites, 1996-1999. *Clinical Infectious Diseases;* **38** (Suppl. 3): S175-S180. |
| 110 | **Ravel A, *et al***. (2011) Description and burden of travel-related cases caused by enteropathogens reported in a Canadian community. *Journal of Travel Medicine*; **18**: 8-19. |
| 111 | **Bless PJ, *et al***. (2017) Time trends of positivity rates from foodborne pathogen testing in Switzerland, 2003 to 2012. *Swiss Medical Weekly*; **147**: w14569. |
| 112 | **Domínguez A, *et al***. (2007) Foodborne *Salmonella*-caused outbreaks in Catalonia (Spain), 1990 to 2003. *Journal of Food Protection;* **70**: 209-213. |
| 113 | **MacDonald E, *et al***. (2018) The role of domestic reservoirs in domestically acquired *Salmonella* infections in Norway: epidemiology of salmonellosis, 2000-2015, and results of a national prospective case-control study, 2010-2012. *Epidemiology and Infection*; **147**: 1-8. |
| 114 | **Daniels NA, *et al***. (2000) *Vibrio parahaemolyticus* infections in the United States, 1973-1998. *The Journal of Infectious Diseases;* **181**: 1661-1666. |
| 115 | **Bassal R, *et al***. (2016) Trends in the epidemiology of campylobacteriosis in Israel (1999-2012). *Foodborne Pathogens and Disease*; **13**: 448-455. |
| 116 | **Flugelman AA, *et al***. (2019) Epidemiologic surveillance in Israel of *Cryptosporidium*, a unique waterborne notifiable pathogen, and public health policy. *Israel Medical Association Journal*; **21**: 589-594. |
| 117 | **Taylor EV, *et al***. (2013) Common source outbreaks of *Campylobacter* infection in the USA, 1997-2008. *Epidemiology and Infection*; **141**: 987-996. |
| 118 | **Frenzen PD**. (2003) Mortality due to gastroenteritis of unknown etiology in the United States. *The Journal of Infectious Diseases;* **187**: 441-452. |
| 119 | **Semenza JC, Nichols G**. (2007) Cryptosporidiosis surveillance and water-borne outbreaks in Europe. *Eurosurveillance*; **12**: E13-E14. |
| 120 | **Jiang Y, *et al***. (2018) *Cyclospora cayetanensis* infections among diarrheal outpatients in Shanghai: a retrospective case study. *Frontiers in Medicine*; **12**: 98-103. |
| 121 | **Milczarek M, *et al***. (2019) Salmonellosis in Poland in 2017. *Przeglad Epidemiologiczny*; **73**: 463-477. |
| 122 | **Stypułkowska-Misiurewicz H, Baumann-Popczyk A**. (2013) Shigellosis in Poland in 2011. *Przeglad Epidemiologiczny*; **67**: 217-219. |
| 123 | **Yoder JS, *et al***. (2012) Cryptosporidiosis surveillance--United States, 2009-2010. *Morbidity and Mortality Weekly Report*; **61**: 1-12. |
| 124 | **Onishi N, *et al***. (2008) Molecular epidemiology of norovirus gastroenteritis in Soma, Japan, 2001-2003. *Pediatrics International*; **50**: 65-69. |
| 125 | **Miettinen IT, *et al***. (2001) Waterborne epidemics in Finland in 1998-1999. *Water Science and Technology*; **43**: 67-71. |
| 126 | **Milczarek M, Czarkowski MP, Sadkowska-Todys M**. (2018) Salmonellosis in Poland in 2015 and 2016. *Przeglad Epidemiologiczny*; **72**: 419-431. |
| 127 | **Iqbal J, *et al***. (2001) Cryptosporidiosis in Kuwaiti children: seasonality and endemicity. *Clinical Microbiology and Infection*; **7**: 261-266. |
| 128 | **Leshem E, *et al***. (2013) Genotype GI.6 norovirus, United States, 2010-2012. *Emerging Infectious Diseases*; **19**: 1317-1320. |
| 129 | **Welby S, *et al***. (2011) Comparison of *Salmonella enteritidis* phage types isolated from layers and humans in Belgium in 2005. *Foodborne Pathogens and Disease*; **8**: 929-934. |
| 130 | **Conteas CN, *et al***. (1998) Examination of the prevalence and seasonal variation of intestinal microsporidiosis in the stools of persons with chronic diarrhea and human immunodeficiency virus infection. *American Journal of Tropical Medicine and Hygiene;* **58**: 559-561. |
| 131 | **Bahk GJ, Kim YS, Park MS**. (2015) Use of Internet search queries to enhance surveillance of foodborne illness. *Emerging Infectious Diseases*; **21**: 1906-1912. |
| 132 | **Poirier E, *et al***. (2008) Evaluation of the impact on human salmonellosis of control measures targeted to *Salmonella enteritidis* and *typhimurium* in poultry breeding using time-series analysis and intervention models in France. *Epidemiology and Infection*; **136**: 1217-1224. |
| 133 | **Uejio CK, *et al***. (2014) Drinking water systems, hydrology, and childhood gastrointestinal illness in Central and Northern Wisconsin. *American Journal of Public Health*; **104**: 639-646. |
| 134 | **Williams MS, *et al***. (2015) Temporal patterns of *Campylobacter* contamination on chicken and their relationship to campylobacteriosis cases in the United States. *International* *Journal of Food Microbiology*; **208**: 114-121. |
| 135 | **Williams MS, *et al***. (2010) Determining relationships between the seasonal occurrence of *Escherichia coli* O157:H7 in live cattle, ground beef, and humans. *Foodborne Pathogens and Disease*; **7**: 1247-1254. |
| 136 | **Varga C, *et al***. (2015) Spatial-temporal epidemiology of human *Salmonella enteritidis* infections with major phage types (PTs 1, 4, 5b, 8, 13, and 13a) in Ontario, Canada, 2008-2009. *BMC Public Health*; **15**: 1247. |
| 137 | **Aik J, *et al***. (2018) Climate variability and salmonellosis in Singapore - a time series analysis. *Science of the Total Environment*; **639**: 1261-1267. |
| 138 | **Lal A, *et al***. (2016) Spatial and temporal variation in the association between temperature and salmonellosis in NZ. *Australian and New Zealand Journal of Public Health*; **40**: 165-169. |
| 139 | **Gradel KO, *et al****.* (2009) Are host characteristics or exposure factors mainly involved in the acquisition of zoonotic *Salmonella* and *Campylobacter* coinfection in humans? *Foodborne Pathogens and Disease*; **6**: 251-255. |
| 140 | **Gabriel E, *et al*.** (2010) Spatio-temporal epidemiology of *Campylobacter jejuni* enteritis, in an area of Northwest England, 2000-2002. *Epidemiology and Infection*; **138**: 1384-1390. |
| 141 | **Sumi A, *et al***. (2009) Predicting the incidence of human campylobacteriosis in Finland with time series analysis. *Acta Pathologica, Microbiologica, et Immunologica Scandinavica*; **117**: 614-622. |
| 142 | **Chui KK, *et al***. (2009) Geographic variations and temporal trends of *Salmonella*-associated hospitalization in the US elderly, 1991-2004: a time series analysis of the impact of HACCP regulation. *BMC Public Health*; **9**: 1-10. |
| 143 | **Venkat A, *et al***. (2019) Spatiotemporal patterns of cholera hospitalization in Vellore, India. *International Journal of Environmental Research and Public Health*; **16**: 4257. |
| 144 | **Kaimi I, Diggle PJ**. (2011) A hierarchical model for real-time monitoring of variation in risk of non-specific gastrointestinal infections. *Epidemiology and Infection*; **139**: 1854-1862. |
| 145 | **Brankston G, *et al***. (2018) Assessing the impact of environmental exposures and *Cryptosporidium* infection in cattle on human incidence of cryptosporidiosis in Southwestern Ontario, Canada. *PLOS One*; **13**: e0196573. |
| 146 | **Jagai JS, *et al***. (2012) Seasonal patterns of gastrointestinal illness and streamflow along the Ohio River. *International Journal of Environmental Research and Public Health*; **9**: 1771-1790. |
| ***Citations Not Referenced in Manuscript Text (in alphabetical order)*** | |
| 147 | **Akil L, Ahmad HA, Reddy RS**. (2014) Effects of climate change on *Salmonella* infections. *Foodborne Pathogens and Disease*; **11**: 974-980. |
| 148 | **Ako AA, Nkeng GE, Takem GE**. (2009) Water quality and occurrence of water-borne diseases in the Douala 4th District, Cameroon. *Water Science and Technology*; **59**: 2321-2329. |
| 149 | **Bi P, *et al***. (2008) Weather and notified *Campylobacter* infections in temperate and sub-tropical regions of Australia: an ecological study. *Journal of Infection*; **57**: 317-323. |
| 150 | **Bonner C, *et al***. (2001) Analysis of outbreaks of infectious intestinal disease in Ireland: 1998 and 1999. *Irish Medical Journal*; **94**: 142-144. |
| 151 | **Boore AL, *et al***. (2015) *Salmonella enterica* infections in the United States and assessment of coefficients of variation: a novel approach to identify epidemiologic characteristics of individual serotypes, 1996-2011. *PLOS One*; **10**: e0145416. |
| 152 | **Buuck S, *et al***. (2020) Epidemiology of enterotoxigenic *Escherichia coli* infection in Minnesota, 2016-2017. *Epidemiology and Infection*; **148**: e206. |
| 153 | **Cha W, *et al***. (2016) Factors associated with increasing campylobacteriosis incidence in Michigan, 2004-2013. *Epidemiology and Infection*; **144**: 3316-3325. |
| 154 | **Chakraborty A, *et al***. (2015) The descriptive epidemiology of yersiniosis: a multistate study, 2005-2011. *Public Health Reports*; **130**: 269-277. |
| 155 | **Chiou CS, *et al***. (2000) *Vibrio parahaemolyticus* serovar O3:K6 as cause of unusually high incidence of food-borne disease outbreaks in Taiwan from 1996 to 1999. *Journal of Clinical Microbiology*; **38**: 4621-4625. |
| 156 | **Craun GF, *et al***. (2010) Causes of outbreaks associated with drinking water in the United States from 1971 to 2006. *Clinical Microbiology Reviews Journal*; **23**: 507-528. |
| 157 | **Curriero FC, *et al***. (2001) The association between extreme precipitation and waterborne disease outbreaks in the United States, 1948-1994. *American Journal of Public Health*; **91**: 1194-1199. |
| 158 | **Davis CA, Vally H, Beard FH**. (2011) Norovirus in residential care facilities: does prompt notification of outbreaks help? *Communicable Diseases Intelligence*; **35**: 162-167. |
| 159 | **Edge VL, *et al***. (2007) Physician diagnostic and reporting practices for gastrointestinal illnesses in three health regions of British Columbia. *Canadian Journal of Public Health*; **98**: 306-310. |
| 160 | **Espenhain L, *et al***. (2019) Epidemiology and impact of norovirus outbreaks in Norwegian healthcare institutions, 2005-2018. *Journal of Hospital Infection*; **103**: 335-340. |
| 161 | **Fagan RP, *et al***. (2011) Endemic foodborne botulism among Alaska Native persons--Alaska, 1947-2007. *Clinical Infectious Diseases*; **52**: 585-592. |
| 162 | **Faustini A, Giorgi Rossi P, Perucci CA**. (2003) Outbreaks of food borne diseases in the Lazio region, Italy: the results of epidemiological field investigations. *European Journal of Epidemiology*; **18**: 699-702. |
| 163 | **Fisher IS, *et al***. (2009) Human infections due to *Salmonella* *Napoli*: A multicountry, emerging enigma recognized by the Enter-net international surveillance network. *Foodborne Pathogens and Disease*; **6**: 613-619. |
| 164 | **Fleury M, *et al***. (2006) A time series analysis of the relationship of ambient temperature and common bacterial enteric infections in two Canadian provinces. *International Journal of Biometeorology*; **50**: 385-391. |
| 165 | **Franklin K, *et al***. (2015) Stool submission data to help inform population-level incidence rates of enteric disease in a Canadian community. *Epidemiology and Infection*; **143**: 1368-1376. |
| 166 | **Galbraith NS, Forbes P, Clifford C**. (1982) Communicable disease associated with milk and dairy products in England and Wales 1951-80. *British Medical Journal*; **284**: 1761-1765. |
| 167 | **Geissler AL, *et al***. (2017) Increasing *Campylobacter* infections, outbreaks, and antimicrobial resistance in the United States, 2004-2012. *Clinical Infectious Diseases*; **65**: 1624-1631. |
| 168 | **Gillespie IA, *et al***. (2005) Foodborne general outbreaks of *Salmonella enteritidis* phage type 4 infection, England and Wales, 1992-2002: where are the risks? *Epidemiology and Infection*; **133**: 795-801. |
| 169 | **Gingold DB, Strickland MJ, Hess JJ.** (2014) Ciguatera fish poisoning and climate change: analysis of National Poison Center data in the United States, 2001-2011. *Environmental Health Perspectives;* **122**: 580-586. |
| 170 | **Hall AJ, *et al***. (2013) Acute gastroenteritis surveillance through the National Outbreak Reporting System, United States. *Emerging Infectious Diseases*; **19**: 1305-1309. |
| 171 | **Hall RL, Jones JL, Herwaldt BL**. (2011) Surveillance for laboratory-confirmed sporadic cases of cyclosporiasis--United States, 1997-2008. *Morbidity and Mortality Weekly Report*; **60**: 1-11. |
| 172 | **Hamplová L, Kotrbová K, Príkazská M**. (2016) Campylobacteriosis in the South Bohemian Region - a recurrent problem. *Epidemiologie, Mikrobiologie, Imunologie*; **65**: 193-197. |
| 173 | **Hearnden M, *et al***. (2003) The regionality of campylobacteriosis seasonality in New Zealand. *International Journal of Environmental Health Research*; **13**: 337-348. |
| 174 | **Hermida RC, Ayala DE, Arróyave RJ**. (1990) Circannual incidence of *Giardia lamblia* in Mexico. *Chronobiology International*; **7**: 329-340. |
| 175 | **Hoque E, *et al***. (2004) A descriptive epidemiology of giardiasis in New Zealand and gaps in surveillance data. *The New Zealand Medical Journal*; **117**: U1149. |
| 176 | **Inglis GD, Teixeira JS, Boras VF**. (2021) Comparative prevalence and diversity of *Campylobacter jejuni* strains in water and human beings over a 1-year period in southwestern Alberta, Canada. *Canadian Journal of Microbiology*. Published online: 7 July 2021. doi:10.1139/cjm-2021-0158. |
| 177 | **Jafari F, *et al***. (2009) Diagnosis and prevalence of enteropathogenic bacteria in children less than 5 years of age with acute diarrhea in Tehran children's hospitals. *Journal of Infection*; **58**: 21-27. |
| 178 | **Jenssen GR, *et al***. (2019) Implementation of multiplex PCR diagnostics for gastrointestinal pathogens linked to increase of notified shiga toxin-producing *Escherichia coli* cases in Norway, 2007-2017. *European Journal of Clinical Microbiology and Infectious Diseases*. Published online: 24 January 2019. doi:10.1007/s10096-019-03475-5. |
| 179 | **Kim BR, *et al***. (2018) Incidence and characteristics of norovirus-associated benign convulsions with mild gastroenteritis, in comparison with rotavirus ones. *Brain and Development*; **40**: 699-706. |
| 180 | **Lee MB, Middleton D**. (2003) Enteric illness in Ontario, Canada, from 1997 to 2001. *Journal of Food Protection;* **66**: 953-961. |
| 181 | **Marcus R, *et al***. (2004) Dramatic decrease in the incidence of *Salmonella* serotype enteritidis infections in 5 FoodNet sites: 1996-1999. *Clinical Infectious Diseases;* **38** (Suppl. 3): S135-S141. |
| 182 | **Marshall KE, *et al***. (2020) Lessons learned from a decade of investigations of shiga toxin-producing *Escherichia coli* outbreaks linked to leafy greens, United States and Canada. *Emerging Infectious Diseases*; **26**: 2319-2328. |
| 183 | **Martínez A, *et al***. (2013) Norovirus: a growing cause of gastroenteritis in Catalonia (Spain)? *Journal of Food Protection;* **76**: 1810-1816. |
| 184 | **Meakins SM, *et al***. (2003) General outbreaks of infectious intestinal disease (IID) in hospitals, England and Wales, 1992-2000. *Journal of Hospital Infection*; **53**: 1-5. |
| 185 | **Moffatt CR, *et al***. (2017) The campylobacteriosis conundrum - examining the incidence of infection with *Campylobacter sp.* in Australia, 1998-2013. *Epidemiology and Infection*; **145**: 839-847. |
| 186 | **Mohamed AS, *et al***. (2014) Temporal patterns of human and canine *Giardia* infection in the United States: 2003-2009. *Preventive Veterinary Medicine*; **113**: 249-256. |
| 187 | **Nesbitt A, *et al***. (2012) Integrated surveillance and potential sources of *Salmonella enteritidis* in human cases in Canada from 2003 to 2009. *Epidemiology and Infection*; **140**: 1757-1772. |
| 188 | **Newton A, *et al***. (2012) Increasing rates of vibriosis in the United States, 1996-2010: review of surveillance data from 2 systems. *Clinical Infectious Diseases;* **54** (Suppl. 5): S391-S395. |
| 189 | **Olsen SJ, *et al***. (2003) Outbreaks of typhoid fever in the United States, 1960-99. *Epidemiology and Infection*; **130**: 13-21. |
| 190 | **Orozco-Mosqueda GE, Martínez-Loya OA, Ortega YR**. (2014) *Cyclospora cayetanensis* in a pediatric hospital in Morelia, México. *The American Journal of Tropical Medicine and Hygiene;* **91**: 537-540. |
| 191 | **Osei-Tutu B, Anto F**. (2016) Trends of reported foodborne diseases at the Ridge Hospital, Accra, Ghana: a retrospective review of routine data from 2009-2013. *BMC Infectious Diseases*; **16**: 139. |
| 192 | **Ostrek J, Baumann-Popczyk A, Sadkowska-Todys M**. (2013) Foodborne infections and intoxications in Poland in 2011. *Przeglad Epidemiologiczny*; **67**: 461-466. |
| 193 | **Pogreba-Brown K, Barrett E**. (2018) Campylobacter and ethnicity-A case-case analysis to determine differences in disease presentation and risk factors. *Foodborne Pathogens and Disease*; **15**: 277-284. |
| 194 | **Pornruangwong S, *et al***. (2011) Epidemiological investigation of *Salmonella enterica* serovar Kedougou in Thailand. *Foodborne Pathogens and Disease*; **8**: 203-211. |
| 195 | **Potter RC, Kaneene JB, Gardiner J**. (2002) A comparison of *Campylobacter jejuni* enteritis incidence rates in high- and low-poultry-density counties: Michigan 1992-1999. *Vector-Borne and Zoonotic Diseases*; **2**: 137-143. |
| 196 | **Powell MR, *et al***. (2018) Temporal patterns in principal *Salmonella* serotypes in the USA; 1996-2014. *Epidemiology and Infection*; **146**: 437-441. |
| 197 | **Purayidathil FW, Ibrahim J**. (2012) A summary of health outcomes: multistate foodborne disease outbreaks in the U.S., 1998-2007. *Journal of Environmental Health*; **75**: 8-13. |
| 198 | **Rambaud L, Galey C, Beaudeau P**. (2016) Automated detection of case clusters of waterborne acute gastroenteritis from health insurance data - pilot study in three French districts. *Journal of Water and Health*; **14**: 306-316. |
| 199 | **Ray SM, *et al***. (2004) Population-based surveillance for *Yersinia enterocolitica* infections in FoodNet sites, 1996-1999: higher risk of disease in infants and minority populations. *Clinical Infectious Diseases;* **38** (Suppl. 3): S181-S189. |
| 200 | **Rind E, Pearce J**. (2010) The spatial distribution of campylobacteriosis in New Zealand, 1997-2005. *Epidemiology and Infection*; **138**: 1359-1371. |
| 201 | **Rosner BM, Stark K, Werber D**. (2010) Epidemiology of reported *Yersinia enterocolitica* infections in Germany, 2001-2008. *BMC Public Health*; **10**: 337. |
| 202 | **Sakon N, *et al***. (2015) Impact of genotype-specific herd immunity on the circulatory dynamism of norovirus: a 10-year longitudinal study of viral acute gastroenteritis. *The Journal of Infectious Diseases;* **211**: 879-888. |
| 203 | **Schmutz C, *et al***. (2016) Inverse trends of *Campylobacter* and *Salmonella* in Swiss surveillance data, 1988-2013. *Eurosurveillance*; **21**: 30130. |
| 204 | **Sharp JC, Paterson GM, Forbes GI**. (1980) Milk-borne salmonellosis in Scotland. *Journal of Infection*; **2**: 333-340. |
| 205 | **Shim JO, *et al***. (2017) Different age distribution between campylobacteriosis and nontyphoidal salmonellosis in hospitalized Korean children with acute inflammatory diarrhea. *Journal of Korean Medical Science*; **32**: 1202-1206. |
| 206 | **Sivapalasingam S, *et al***. (2004) *Salmonella* bacteriuria: an increasing entity in elderly women in the United States. *Epidemiology and Infection*; **132**: 897-902. |
| 207 | **Soltan Dallal MM, *et al***. (2020) Associations between climatic parameters and the human salmonellosis in Yazd province, Iran. *Environmental Research*; **187**: 109706. |
| 208 | **Steele MK, *et al***. (2020) Characterizing Norovirus transmission from outbreak data, United States. *Emerging Infectious Diseases*; **26**: 1818-1825. |
| 209 | **Syue LS, *et al***. (2016) Monomicrobial Aeromonas and Vibrio bacteremia in cirrhotic adults in southern Taiwan: Similarities and differences. *Journal of Microbiology, Immunology, and Infection*; **49**: 509-515. |
| 210 | **Thomas KM, *et al***. (2006) A role of high impact weather events in waterborne disease outbreaks in Canada, 1975 - 2001. *International Journal of Environmental Health Research*; **16**: 167-180. |
| 211 | **Todd ECD**. (1992) Foodborne Disease in Canada - a 10-year Summary from 1975 to 1984. *Journal of Food Protection;* **55**: 123-132. |
| 212 | **Unicomb LE, *et al***. (2009) Outbreaks of campylobacteriosis in Australia, 2001 to 2006. *Foodborne Pathogens and Disease*; **6**: 1241-1250. |
| 213 | **Venuto M, *et al***. (2010) Analyses of the eFORS (Electronic Foodborne Outbreak Reporting System) surveillance data (2000-2004) in school settings. *Journal of Environmental Health*; **72**: 8-13. |
| 214 | **Watier L, Richardson S, Hubert B**. (1993) *Salmonella enteritidis* infections in France and the United States: characterization by a deterministic model. *American Journal of Public Health*; **83**: 1694-1700. |
| 215 | **Wikswo ME, Hall AJ**. (2012) Outbreaks of acute gastroenteritis transmitted by person-to-person contact--United States, 2009-2010. *Morbidity and Mortality Weekly Report*; **61**: 1-12. |
| 216 | **Wilson E**. (2015) Foodborne illness and seasonality related to mobile food sources at festivals and group gatherings in the state of Georgia. *Journal of Environmental Health*; **77**: 8-11. |
| 217 | **Woodward WE, *et al***. (1970) Foodborne disease surveillance in the United States, 1966 and 1967. *American Journal of Public Health and the Nation's Health*; **60**: 130-137. |
| 218 | **Yang X, *et al***. (2017) Nontyphoidal *Salmonella* gastroenteritis in Baoshan, Shanghai, China, 2010 to 2014: an etiological surveillance and case-control study. *Journal of Food Protection;* **80**: 482-487. |
| 219 | **Yoshikura H**. (2020) Declining *Vibrio parahaemolyticus* and *Salmonella*, increasing *Campylobacter* and persisting norovirus food poisonings: inference derived from food poisoning statistics of Japan. *Japanese Journal of Infectious Diseases;* **73**: 102-110. |
| 220 | **Zintl A, *et al***. (2009) The prevalence of *Cryptosporidium* species and subtypes in human faecal samples in Ireland. *Epidemiology and Infection*; **137**: 270-277. |

**Supplemental Table S2.** Studies detecting seasonality and estimating peak timing using comparisons between 2 discrete seasons. Season lengths were either equal (6-month intervals) or unequal (often 3-month vs. 9-month seasons). Studies used incidence (i.e., semester divisions, spring/summer vs. fall/winter, high/low incidence) or environmental characteristics (i.e., wet/dry, warm/cool) to define seasons. Studies detected seasonality and estimated peak timing by identifying 1 season with higher average incidence or cumulative infections. Studies either did not conduct formal statistical comparison tests (denoted ‘none’) or compared seasons using formal statistical tests and odds ratio (OR) or incidence rate ratio (IRR) measures of association. Studies assessed either illnesses or outbreaks.

| **Definition of Season** | **Season Lengths** | **Study Years** | **How Seasonality Modeled** | **Statistical Tests Used** | **Outcome** | **Pathogen(s)** | **Country** |
| --- | --- | --- | --- | --- | --- | --- | --- |
| **Based on Incidence** |  |  |  |  |  |  |  |
| First Semester (January-June) vs. Second Semester (July-December) | Equal | 9 | Summary statistics by season | None | Outbreaks | *Cryptosporidium* | England and Wales |
| Spring/Summer (April-September) vs. Fall/Winter (October-March) | Equal | 5 | Summary statistics by season | Mann-Whitney rank-sum test | Outbreaks | *Norovirus* | United States |
| High Intensity (May-October) vs. Low Intensity (November-April) | Equal | 8 | Regression with binary variable | IRR | Illnesses | *Campylobacter* | United States |
| High Intensity (October-March) vs. Low Intensity (April-September) | Equal | 15 | Summary statistics by season | None | Illnesses | *Norovirus* | Japan |
| High Intensity (June-November) vs. Low Intensity (December-May) | Equal | 32 | Regression with binary variable | OR | Illnesses | *Vibrio* | Global |
| High Intensity (December-March) vs. Low Intensity (April-November) | Unequal | 6 | Regression with binary variable | OR | Illnesses | Gastroenteritis | United States |
| **Based on Other Characteristics** |  |  |  |  |  |  |  |
| Wet (April-September) vs.  Dry (October-March) | Equal | 6 | Regression with binary variable | OR | Illnesses | Rotavirus | Bangladesh |
| Wet (November-April) vs.  Dry (May-October) | Equal | 11 | Summary statistics by season | Χ^2^ test | Illnesses | *Campylobacter* | Malawi |
| Wet (October-March) vs.  Dry (April-September) | Equal | 13 | Regression with binary variable | OR | Illnesses | *Cryptosporidium; Giardia* | Canada |
| Wet (June-November) vs.  Dry (December-May) | Equal | 21 | Summary statistics by season | None | Illnesses | *Vibrio* | Bangladesh |
| Wet (July-October) vs.  Dry (November-June) | Unequal | 7 | Summary statistics by season | None | Illnesses | *Salmonella* | Thailand |
| Wet (June-August) vs.  Dry (September-May) | Unequal | 10 | Summary statistics by season | None | Illnesses | *Cyclospora* | Mexico |
| Wet (November-March) vs.  Dry (April-October) | Unequal | 10 | Summary statistics by season | Student’s t-test | Illnesses | Gastroenteritis | Botswana |
| Wet (June-September) vs.  Dry (October-May) | Unequal | 13 | Regression with binary variable | OR | Illnesses | *Salmonella* | India |
| Warm (May-October) vs.  Cool (November-April) | Equal | 7 | Summary statistics by season | None | Outbreaks | *Salmonella* | Japan |
| Warm (May-October) vs.  Cool (November-April) | Equal | 11 | Summary statistics by season | Χ^2^ test | Outbreaks | *Norovirus; Salmonella* | Spain |
| Warm vs. Cool  (Dates varied by study year) | Unequal | 23 | Regression with binary variable | IRR | Illnesses | *Salmonella* | Australia |
| Warm & Dry (December-March, July-August, December) vs.  Cool & Wet (April-June, September-November) | Equal | 4 | Regression with binary variable | OR | Illnesses | Rotavirus | Kenya |

**Supplemental Table S3.** A summary of studies detecting seasonality and estimating peak timing using comparisons between 4 discrete seasons. Studies often defined seasons using quarterly divisions named summer, fall, winter, and spring. We provide the definition of the highest-incidence season, often described as summer, in chronological order according to calendar month to illustrate differences in definitions by geographic location. Studies estimated peak timing by identifying the season with the highest average or median incidence or cumulative infections compared to other seasons. Studies either did not conduct formal statistical comparison tests (denoted ‘none’) or compared seasons using formal statistical tests and odds ratio (OR) or incidence rate ratio (IRR) measures of association. Studies assessed health outcomes including illnesses, hospitalizations, and outbreaks.

| **Definition of Summer or Highest-Incidence Season** | **Study Years** | **How Seasonality Modeled** | **Statistical Tests Used** | **Outcome(s)** | **Pathogen(s)** | **Country** |
| --- | --- | --- | --- | --- | --- | --- |
| December 1^st^–February 1^st^ | 26 | Regression with indicator variable | IRR | Illnesses | *Campylobacter* | Australia |
| December-February | 8 | Principal component analysis | Variable loadings | Illnesses | *Campylobacter* | New Zealand |
| December-February | 16 | Regression with indicator variable | IRR | Illnesses | *Campylobacter* | Australia |
| December-February | 20 | Summary statistics by season | None | Hospitalizations | *Campylobacter* | New Zealand |
| January-March | 3 | Summary statistics by season | None | Outbreaks | *Salmonella* | Brazil |
| April-June | 9 | Summary statistics by season | None | Outbreaks | Gastroenteritis | England and Wales |
| June-August | 1 | Summary statistics by season | None | Illnesses | *Campylobacter; E. coli; Salmonella; Shigella; Vibrio; Yersinia; Listeria* | United States |
| June-August | 1 | Summary statistics by season | None | Illnesses | *Campylobacter; E. coli; Salmonella; Shigella; Vibrio; Yersinia; Listeria* | United States |
| June-August | 1 | Summary statistics by season | None | Illnesses | *Campylobacter; E. coli; Salmonella; Shigella; Vibrio; Yersinia; Listeria* | United States |
| June-August | 1 | Summary statistics by season | None | Illnesses | *Campylobacter; E. coli; Salmonella; Vibrio; Yersinia; Listeria; Cyclospora; Shigella; Cryptosporidium* | United States |
| June-August | 1 | Summary statistics by season | None | Outbreaks | *Norovirus* | Ireland |
| June-August | 2 | Summary statistics by season | None | Illnesses | *E. coli* | United States |
| June-August | 2 | Summary statistics by season | None | Outbreaks | Gastroenteritis | China |
| June-August | 3 | Summary statistics by season | Χ^2^ test | Illnesses | *Salmonella* | United States |
| June-August | 4 | Summary statistics by season | None | Outbreaks | *Norovirus* | China |
| June-August | 4 | Regression with indicator variable | OR | Outbreaks | *E. coli* | United States |
| June-August | 6 | Summary statistics by season | None | Illnesses | *Salmonella* | United States |
| June-August | 6 | Summary statistics by season | None | Outbreaks | *Campylobacter* | Australia |
| June-August | 6 | Regression with indicator variable | OR | Illnesses | *Salmonella* | Thailand |
| June-August | 7 | Regression with indicator variable | OR | Illnesses | *Listeria* | England and Wales |
| June-August | 7 | Regression with indicator variable | IRR | Illnesses | *Salmonella* | United States |
| June-August | 8 | Summary statistics by season | Χ^2^ test | Illnesses | *E. coli* | United States |
| June-August | 9 | Summary statistics by season | None | Hospitalizations | *Norovirus*; Rotavirus | Republic of Korea |
| June-August | 9 | Regression with indicator variable | OR | Outbreaks | *Norovirus* | United States |
| June-August | 9 | Regression with indicator variable | IRR | Illnesses | *Salmonella; Campylobacter; E. coli* | Canada |
| June-August | 9 | Regression with indicator variable | IRR | Illnesses; Outbreaks | *Campylobacter* | United States |
| June-August | 10 | Summary statistics by season | None | Outbreaks | *E. coli* | United States; Canada |
| June-August | 10 | Summary statistics by season | None | Illnesses | *E. coli* | South Korea |
| June-August | 10 | Regression with indicator variable | OR | Illnesses | *Campylobacter* | United States |
| June-August | 11 | Summary statistics by season | Χ^2^ test | Outbreaks | *Salmonella* | England and Wales |
| June-August | 11 | Regression with indicator variable | IRR | Illnesses | *Campylobacter* | United States |
| June-August | 13 | Summary statistics by season | None | Outbreaks | Gastroenteritis | United States |
| June-August | 14 | Summary statistics by season | None | Illnesses | *Yersinia* | United States |
| June-August | 15 | Summary statistics by season | None | Outbreaks | Gastroenteritis | Denmark; Finland; Norway; Sweden |
| June-August | 17 | Summary statistics by season | Χ^2^ test | Outbreaks | *E. coli* | United States |
| June-August | 19 | Summary statistics by season | None | Illnesses | *Campylobacter* | United States |
| June-August | 26 | Summary statistics by season | None | Illnesses | *Salmonella* | United States |
| June-August | 26 | Summary statistics by season | None | Illnesses | *Campylobacter; Salmonella* | Switzerland |
| June-August | 36 | Summary statistics by season | Χ^2^ test | Illnesses; Outbreaks | Gastroenteritis | United States |
| June-August | 47 | Summary statistics by season | None | Outbreaks | Gastroenteritis | United States |
| June-September | 8 | Random forest classifier score | Random Forest | Outbreaks | *Vibrio* | India |
| June 15th-September 14th | 21 | Summary statistics by season | None | Illnesses | *Salmonella* | United States |
| July-September | 1 | Summary statistics by season | None | Outbreaks | Gastroenteritis | Poland |
| June-September | 3 | Summary statistics by season | None | Illnesses | *Campylobacter; E. coli; Listeria; Salmonella; Shigella; Yersinia* | United States |
| June-September | 3 | Summary statistics by season | None | Illnesses | Gastroenteritis | United States |
| July-September | 3 | Regression with indicator variable | OR | Illnesses | *Vibrio* | United States |
| July-September | 4 | Summary statistics by season | None | Illnesses | *Shigella* | United States |
| July-September | 7 | Regression with indicator variable | OR | Illnesses | *Yersinia* | United States |
| July-September | 7 | Regression with indicator variable | IRR | Illnesses | *Salmonella* | Canada |
| July-September | 10 | Summary statistics by season | None | Illnesses | *Salmonella* | United States |
| July-September | 10 | Summary statistics by season | ANOVA | Illnesses | *Salmonella* | United States |
| July-October | 3 | Summary statistics by season | None | Illnesses | *Giardia* | United States |
| July-October | 3 | Summary statistics by season | None | Illnesses | *Giardia* | United States |
| Not Specified | 1 | Summary statistics by season | None | Illnesses | *Campylobacter* | Canada |
| Not Specified | 2 | Summary statistics by season | None | Illnesses | Gastroenteritis | Canada |
| Not Specified | 2 | Summary statistics by season | None | Illnesses | *Salmonella* | Canada |
| Not Specified | 2 | Summary statistics by season | Χ^2^ test | Illnesses | Gastroenteritis | Iran |
| Not Specified | 2 | Summary statistics by season | None | Outbreaks | Gastroenteritis | Ireland |
| Not Specified | 2 | Summary statistics by season | None | Outbreaks | Gastroenteritis | United States |
| Not Specified | 2 | Summary statistics by season | None | Outbreaks | *Shigella; Salmonella; Norovirus; E. coli* | United States |
| Not Specified | 4 | Summary statistics by season | None | Illnesses | *Giardia* | New Zealand |
| Not Specified | 4 | Summary statistics by season | None | Illnesses | *Salmonella* | United States |
| Not Specified | 4 | Regression with indicator variable | IRR | Illnesses | *Salmonella* | Iran |
| Not Specified | 4 | Summary statistics by season | None | Outbreaks | *Vibrio* | Taiwan |
| Not Specified | 5 | Summary statistics by season | None | Outbreaks | *Norovirus* | European Union |
| Not Specified | 5 | Summary statistics by season | None | Outbreaks | *Norovirus* | United States |
| Not Specified | 6 | Summary statistics by season | Χ^2^ test | Illnesses | *Campylobacter; Salmonella; E. coli* | Canada |
| Not Specified | 8 | Regression with indicator variable | IRR | Illnesses | *Campylobacter* | United States |
| Not Specified | 10 | Summary statistics by season | None | Illnesses; Outbreaks | Gastroenteritis | Canada |
| Not Specified | 10 | Regression with indicator variable | IRR | Illnesses | *Campylobacter* | Czech Republic |
| Not Specified | 10 | Summary statistics by season | None | Outbreaks | *Norovirus* | Japan |
| Not Specified | 11 | Regression with indicator variable | IRR | Illnesses | Ciguatera fish poisoning | United States |
| Not Specified | 16 | Summary statistics by season | None | Illnesses | *Norovirus* | South Korea |
| Not Specified | 18 | Regression with indicator variable | IRR | Illnesses | *Salmonella* | United States |
| Not Specified | 19 | Regression with indicator variable | IRR | Illnesses | *Salmonella* | United States |
| Not Specified | 30 | Summary statistics by season | None | Outbreaks | Gastroenteritis | United States |

**Supplemental Table S4.** A summary of studies detecting seasonality and estimating peak timing by comparing discrete monthly records. We report studies by increasing time series length, which varied from 1-61 years. Studies estimated peak timing by identifying the month with the highest average or median incidence or cumulative infections. Most studies did not formally compare calendar months when examining peak timing. Studies either did not conduct formal statistical comparison tests (denoted ‘none’) or compared seasons using formal statistical tests and odds ratio (OR) or incidence rate ratio (IRR) measures of association. Studies assessed numerous health outcomes including tests, illnesses, per cent positives, hospitalizations, outbreaks, and deaths.

| **Study Years** | **How Seasonality Modeled** | **Statistical Tests Used** | **Outcome(s)** | **Pathogen(s)** | **Country** |
| --- | --- | --- | --- | --- | --- |
| 1 | Summary statistics by season | None | Illnesses | *Cryptosporidium* | European Union |
| 1 | Summary statistics by season | None | Illnesses | *Salmonella* | China |
| 1 | Summary statistics by season | None | Illnesses | *Salmonella* | China |
| 1 | Summary statistics by season | None | Illnesses | *Salmonella* | Poland |
| 1 | Summary statistics by season | None | Illnesses | *Shigellosis* | Poland |
| 1 | Summary statistics by season | None | Illnesses | *Salmonella* | Belgium |
| 1 | Summary statistics by season | None | Illnesses; Outbreaks | Gastroenteritis | United States |
| 1 | Summary statistics by season | None | Illnesses; Outbreaks | *Salmonella* | Belgium |
| 1 | Summary statistics by season | None | Hospitalizations | *Cyclospora* | China |
| 1 | Summary statistics by season | None | Outbreaks | Gastroenteritis | United States |
| 2 | Summary statistics by season | None | Illnesses | *Cryptosporidium* | United States |
| 2 | Summary statistics by season | None | Illnesses | *Giardia* | United States |
| 2 | Summary statistics by season | None | Illnesses | *Giardia* | United States |
| 2 | Summary statistics by season | None | Illnesses | *Norovirus* | Japan |
| 2 | Summary statistics by season | None | Illnesses | *Salmonella* | Poland |
| 2 | Summary statistics by season | None | Outbreaks | *Norovirus; Campylobacter* | Finland |
| 3 | Summary statistics by season | None | Illnesses | *Cryptosporidium* | England |
| 3 | Summary statistics by season | None | Illnesses | *Cryptosporidium* | Kuwait |
| 3 | Summary statistics by season | None | Illnesses | *Cryptosporidium* | United States |
| 3 | Summary statistics by season | None | Illnesses | *Cryptosporidium* | United States |
| 3 | Summary statistics by season | None | Illnesses | *Cryptosporidium* | United States |
| 3 | Summary statistics by season | None | Illnesses | *Cryptosporidium* | United States |
| 3 | Regression with indicator variable | OR | Illnesses | *Salmonella* | Canada |
| 3 | Summary statistics by season | None | Deaths | Gastroenteritis | United States |
| 3 | Summary statistics by season | None | Outbreaks | *Norovirus* | United States |
| 4 | Summary statistics by season | None | Illnesses | *Campylobacter* | United States |
| 4 | Summary statistics by season | None | Illnesses | *Campylobacter* | United States |
| 4 | Summary statistics by season | None | Illnesses | *Yersinia* | United States |
| 4 | Regression with indicator variable | OR | Illnesses | Gastroenteritis | Canada |
| 4 | Summary statistics by season | None | Illnesses; Outbreaks | Gastroenteritis | Australia |
| 4 | Summary statistics by season | None | Outbreaks | Gastroenteritis | France |
| 4 | Summary statistics by season | Mann-Whitney rank-sum test | Positivity Rates | Microsporidia | United States |
| 5 | Summary statistics by season | None | Illnesses | *Campylobacter; Salmonella; E. coli; Shigella; Yersinia; Listeria*; Hepatitis A; *Clostridium* | Canada |
| 5 | Summary statistics by season | None | Illnesses | *Salmonella* | China |
| 5 | Summary statistics by season | None | Illnesses | *Vibrio* | Ghana |
| 5 | Summary statistics by season | None | Illnesses; Outbreaks | *Cyclospora* | United States |
| 5 | Summary statistics by season | None | Outbreaks | *Calicivirus; Norovirus* | United States |
| 5 | Summary statistics by season | None | Outbreaks | Gastroenteritis | Italy |
| 6 | Summary statistics by season | None | Illnesses | *Campylobacter; Salmonella* | South Korea |
| 7 | Summary statistics by season | None | Illnesses | *Cronobacter* | United States |
| 7 | Summary statistics by season | None | Illnesses | *Salmonella* | Italy; France; Switzerland |
| 7 | Summary statistics by season | None | Illnesses | *Salmonella* | United States |
| 8 | Summary statistics by season | None | Illnesses | *Cryptosporidium* | Ireland |
| 8 | Summary statistics by season | None | Illnesses | *Yersinia* | Germany |
| 9 | Summary statistics by season | None | Illnesses | *Salmonella* | Canada |
| 9 | Summary statistics by season | None | Outbreaks | Gastroenteritis | England and Wales |
| 9 | Summary statistics by season | None | Outbreaks | *Norovirus* | England and Wales |
| 10 | Regression with indicator variable | OR | Tests; Per cent Positives | *Campylobacter; Salmonella* | Switzerland |
| 10 | Summary statistics by season | None | Illnesses | *Vibrio* | Taiwan |
| 10 | Summary statistics by season | None | Outbreaks | Gastroenteritis | United States |
| 10 | Summary statistics by season | None | Outbreaks | *Salmonella* | Scotland |
| 11 | Summary statistics by season | None | Illnesses | *Salmonella* | United States |
| 11 | Regression with indicator variable | IRR | Illnesses | *Salmonella* | Australia |
| 12 | Summary statistics by season | None | Illnesses | *Cyclospora* | United States |
| 12 | Summary statistics by season | None | Illnesses | Gastroenteritis | Cameroon |
| 12 | Summary statistics by season | None | Outbreaks | *Campylobacter* | United States |
| 13 | Summary statistics by season | None | Illnesses | *Cryptosporidium* | United States |
| 13 | Summary statistics by season | None | Illnesses | *Cyclospora* | United States |
| 13 | Summary statistics by season | None | Illnesses; Outbreaks | *Giardia* | United States |
| 13 | Summary statistics by season | None | Outbreaks | *Clostridium* | United States |
| 14 | Summary statistics by season | None | Illnesses | *Campylobacter* | Israel |
| 14 | Summary statistics by season | None | Illnesses | *Cryptosporidium* | Israel |
| 14 | Regression with indicator variable | IRR | Illnesses; Outbreaks; Hospitalizations; Deaths | *Salmonella* | Spain |
| 15 | Summary statistics by season | None | Illnesses | *Vibrio* | United States |
| 15 | Summary statistics by season | None | Outbreaks | Gastroenteritis | South Korea; Japan |
| 16 | Summary statistics by season | None | Illnesses | *Campylobacter* | Denmark |
| 16 | Regression with indicator variable | IRR | Illnesses | *Salmonella* | Norway |
| 16 | Summary statistics by season | None | Outbreaks | Gastroenteritis | United States |
| 18 | Summary statistics by season | None | Illnesses; Hospitalizations | *Salmonella* | Poland |
| 19 | Summary statistics by season | None | Outbreaks | Gastroenteritis | Japan |
| 20 | Summary statistics by season | None | Illnesses | *Salmonella* | United States |
| 20 | Summary statistics by season | None | Outbreaks | *Norovirus* | Japan |
| 26 | Summary statistics by season | None | Illnesses | *Vibrio* | United States |
| 26 | Summary statistics by season | None | Deaths | *Listeriosis* | United States |
| 27 | Summary statistics by season | None | Outbreaks | Gastroenteritis | Canada |
| 30 | Summary statistics by season | None | Outbreaks | Gastroenteritis | England and Wales |
| 40 | Summary statistics by season | None | Outbreaks | *Salmonella* | United States |
| 44 | Summary statistics by season | None | Illnesses | Brucellosis | Germany |
| 61 | Summary statistics by season | None | Outbreaks | Botulism | United States |

**Supplemental Table S5.** A summary of studies describing seasonality as a continuous process where seasonal curves were modeled using: i) moving average and kernel density smoothers; ii) cubic splines; iii) seasonal trend decomposition (STL); iv) seasonal autoregressive integrated moving average (SARIMA) models; v) Fourier series and harmonic logistic regression models; and vi) spectral analyses. The temporal resolution data ranged from daily to monthly time units. Studies assessed a variety of health outcomes including illnesses, hospitalizations, and outbreaks.

| **How Data Modeled** | **How Seasonality Modeled** | **Study Years** | **Temporal Resolution** | **Outcome(s)** | **Pathogen(s)** | **Country** |
| --- | --- | --- | --- | --- | --- | --- |
| Poisson regression model with cubic spline | Visual inspection | 9 | Weekly | Illnesses | Gastroenteritis | United States |
| Poisson regression model with cubic spline | Visual inspection | 9 | Monthly | Illnesses | *Cryptosporidium* | Australia |
| Poisson regression model with cubic spline | Visual inspection | 10 | Daily | Illnesses | *Campylobacter* | England and Wales |
| Poisson regression model with cubic spline | Visual inspection | 22 | Monthly | Illnesses | *Vibrio* | Nigeria |
| Moving average smoother | Visual inspection | 2 | Monthly | Illnesses | *Salmonella* | Canada |
| Kernel density smoother | Visual inspection | 9 | Weekly | Illnesses | *Campylobacter* | European Union |
| Seasonal Trend Decomposition | Visual Inspection | 8 | Monthly | Illnesses | *Campylobacter* | United States |
| Seasonal Trend Decomposition | Visual Inspection | 9 | Monthly | Illnesses | *E. coli.* | United States |
| ARIMA regression | Seasonal autoregressive model parameters | 3 | Monthly | Illnesses | Gastroenteritis | South Korea |
| ARIMA regression | Seasonal autoregressive model parameters | 5 | Monthly | Hospitalizations | Gastroenteritis | South Korea |
| ARIMA regression | Seasonal autoregressive model parameters | 7 | Monthly | Illnesses | *Giardia* | United States |
| ARIMA regression | Seasonal autoregressive model parameters | 10 | Daily | Illnesses | *Salmonella* | Australia |
| ARIMA regression | Seasonal autoregressive model parameters | 11 | Monthly | Illnesses | *Vibrio* | India |
| ARIMA regression | Seasonal autoregressive model parameters | 12 | Monthly | Illnesses | *Salmonella* | France |
| ARIMA regression | Seasonal autoregressive model parameters | 12 | Monthly | Outbreaks | *Vibrio* | Taiwan |
| Fourier series | Significant harmonic term coefficients | 3 | Daily | Illnesses | Gastroenteritis | England |
| Fourier series | Significant harmonic term coefficients | 5 | Monthly | Illnesses | *Campylobacter* | Canada |
| Fourier series | Significant harmonic term coefficients | 10 | Daily | Illnesses | Rotavirus | Bangladesh, Brazil, India, Nepal, Pakistan, Peru, Tanzania, South Africa |
| Fourier series | Significant harmonic term coefficients | 11 | Monthly | Illnesses | *Campylobacter* | United Kingdom |
| Fourier series | Significant harmonic term coefficients | 20 | Weekly | Illnesses | *Salmonella* | European Union |
| Fourier series | Application of the δ-methods | 14 | Weekly | Illnesses | *Campylobacter* | United States |
| Linear regression model | Significant harmonic term coefficients | 2 | Monthly | Illnesses | *Salmonella* | France; United States |
| Linear regression model | Significant harmonic term coefficients | 9 | Monthly | Illnesses | *Giardia* | Mexico |
| Negative binomial regression model | Significant harmonic term coefficients | 10 | Monthly | Illnesses | *Salmonella* | United States |
| Negative binomial regression model | Significant harmonic term coefficients | 11 | Daily | Illnesses | *E. coli* | Norway |
| Negative binomial regression model | Significant harmonic term coefficients | 11 | Weekly | Illnesses | *Salmonella* | Singapore |
| Negative binomial regression model | Significant harmonic term coefficients | 18 | Monthly | Illnesses | *Vibrio* | United States |
| Negative binomial regression model | Application of the δ-methods | 6 | Monthly | Illnesses | Rotavirus | Costa Rica |
| Negative binomial regression model | Application of the δ-methods | 8 | Daily | Illnesses | *Salmonella* | Russia |
| Negative binomial regression model | Application of the δ-methods | 16 | Weekly | Illnesses | *Vibrio* | India |
| Negative binomial regression model | Application of the δ-methods | 22 | Monthly | Illnesses | *Campylobacter; Salmonella; Cryptosporidium; E. coli; Shigella; Yersinia; Vibrio; Listeria; Cyclospora* | United States |
| Poisson regression model | Application of the δ-methods | 2 | Weekly | Illnesses | Gastroenteritis | India |
| Poisson regression model | Significant harmonic term coefficients | 4 | Daily | Illnesses | *Vibrio* | Haiti |
| Poisson regression model | Significant harmonic term coefficients | 3 | Daily | Illnesses | *Campylobacter* | England |
| Poisson regression model | Significant harmonic term coefficients | 6 | Monthly | Illnesses | *Cryptosporidium* | Canada |
| Poisson regression model | Significant harmonic term coefficients | 11 | Weekly | Illnesses | *Salmonella* | New Zealand |
| Poisson regression model | Significant harmonic term coefficients | 12 | Weekly | Illnesses | *Campylobacter* | Israel |
| Poisson regression model | Significant harmonic term coefficients | 13 | Daily | Illnesses | *Salmonella; Campylobacter* | Denmark |
| Poisson regression model | Application of the δ-methods | 2 | Weekly | Illnesses | Gastroenteritis | India |
| Poisson regression model | Application of the δ-methods | 7 | Daily | Illnesses | Rotavirus | Russia |
| Poisson regression model | Application of the δ-methods | 10 | Daily | Illnesses | *Giardia; Cryptosporidium; Salmonella; Campylobacter; Shigella;* Hepatitis A | United States |
| Poisson regression model | Application of the δ-methods | 14 | Weekly | Hospitalizations | Gastroenteritis | United States |
| Poisson regression model | Application of the δ-methods | 14 | Weekly | Hospitalizations | Gastroenteritis | United States |
| Poisson regression model | Application of the δ-methods | 14 | Weekly | Hospitalizations | *Salmonella* | United States |
| Poisson regression model | Application of the δ-methods | 14 | Weekly | Hospitalizations | *Salmonella* | United States |
| Poisson regression model | Application of the δ-methods | 14 | Weekly | Hospitalizations | *Salmonella* | United States |
| Spectral analysis | Significant harmonic term coefficients | 6 | Monthly | Illnesses | *Campylobacter* | Finland |
